# Fluorescent-Conjugated ZnO Nanostructures Exhibited 3D Anti-Tumor Efficacy Against Drug-Resistant Cancers Through Cholesterol-Mediated ROS Regulation

**DOI:** 10.3390/antiox15080935

**Published:** 2026-07-28

**Authors:** Salida Ali, Yu Li, Ontana Yotnarong, Ruofan Shi, Ruochen Ma, Chi Yao, Xiaohao Ruan, Jingyi Huang, Da Huang, Yongle Zhan, Theeranan Tangthong, Rong Na

**Affiliations:** 1 Division of Urology, Department of Surgery, School of Clinical Medicine, LKS Faculty of Medicine, The University of Hong Kong, Hong Kong 999077, Chinaruochenm@connect.hku.hk (R.M.);; 2 Department of Otolaryngology-Head and Neck Surgery, The Eighth Affiliated Hospital, Sun Yat-Sen University, Shenzhen 518033, China; litianshun1986@163.com; 3 Nuclear Technology Research and Development Center, Thailand Institute of Nuclear Technology (Public Organization), Nakhon Nayok 26120, Thailand; ontanayr@gmail.com; 4 Department of Urology, Ruijin Hospital, Shanghai Jiao Tong University School of Medicine, Shanghai 200025, Chinavincentruan96@gmail.com (X.R.);

**Keywords:** ZnO nanoparticles, hormone-related cancers, prostate cancer, thyroid cancer, *Camellia sinensis*, cholesterol, reactive oxygen species

## Abstract

ZnO nanoparticles (ZnO NPs) have been widely investigated in the biomedical field, particularly their anti-tumor efficacy. The potential of ZnO hierarchical structures (ZnO HSs) in tumor cell eradication remains largely unexplored in prostate cancer (PCa) and thyroid cancer (TC). In this study, we successfully synthesized and characterized ZnO NPs and ZnO HSs using green tea extract (*Camellia sinensis*) as a reducing agent and conjugation of FIT-C tracking for both ZnO NPs and ZnO HSs. UV-vis spectrophotometry, Dynamic Light Scattering (DLS), FTIR, XDR, SEM and TEM revealed significant differences in morphology between ZnO NPs and ZnO HSs. Our in vitro experiments demonstrated that SNPs were more effective on aggressive PCa and TC cell lines compared to ZnO NPs. Notably, ZnO HSs exhibited enhanced cytotoxicity in 3D tumor cell spheroid models. Mechanistically, ZnO HSs induced apoptosis through cholesterol-mediated reactive oxygen species (ROS) generation. Our in vivo study revealed no histopathological changes in major organs (liver, kidneys, spleen and lungs), emphasizing the safe administration of both ZnO NPs and ZnO HSs. Our study synthesized FITC-conjugated non-spherical ZnO nanoparticles, providing evidence for a novel treatment strategy for hormone-related cancers and prospective fluorescent-guided nanomedicine.

## 1. Introduction

Metal oxide nanoparticles, particularly zinc oxide nanoparticles (ZnO NPs), play an important role in various biomedical applications, including drug delivery, imaging, and biosensors [1,2,3,4]. Previous studies have revealed that ZnO NPs exhibit specific toxicity towards tumor cells while sparing normal cells, making them promising candidates for intrinsic anti-tumor therapies [5,6,7,8,9]. Because of their distinct pH-dependent solubility, electrostatic surface charge, and capacity to take advantage of the metabolic distinctions between cancerous and healthy tissues, zinc oxide nanoparticles (ZnO NPs) show specific toxicity toward tumor cells [10]. Among the various forms of ZnO NPs, ZnO hierarchical structures (ZnO HSs) possess a unique hierarchical morphology, which offers unique properties and applications due to their larger surface area, which potentially enhances their effectiveness in biomedical uses [11]. Green-labeled nanoparticles are widely used as trackers for tumor cellular uptake in drug delivery or diagnostic purposes [12,13].

Nanomaterials have revolutionized oncology through their highly tunable physicochemical properties and targeted delivery capabilities [14]. Among the various metal oxide frameworks, zinc oxide (ZnO) nanostructures have emerged as potent therapeutic agents by demonstrating a unique, tumor-specific cytotoxicity primarily driven by the disruption of intracellular redox homeostasis and the subsequent induction of reactive oxygen species (ROS) [15]. While conventional spherical ZnO nanoparticles (ZnO NPs) are well-characterized—particularly regarding their ability to trigger caspase-3 activation and DNA fragmentation—research is increasingly pivoting toward anisotropic, non-spherical geometries [16].

The shift toward morphological complexity aims to overcome the biological barriers that limit the efficacy of standard spherical carriers. ZnO hierarchical structures (ZnO HSs) offer distinct biomechanical advantages over their spherical counterparts. Their high-aspect-ratio topography facilitates enhanced Membrane Interaction with sharp surfaces, promoting multivalent interactions with deformable cancer cell membranes, significantly lowering the energetic barrier for endocytosis [11]. The “hierarchical structures” architecture allows for localized membrane tension, aiding in direct internalization and cellular uptake. An increased surface-area-to-volume ratio and a higher density of reactive facets accelerate the intracellular release of Zn^2+^ ions.

The synthesis of ZnO NPs/SNPs from green tea extract (*Camellia sinensis*) emphasizes the polyphenolic compounds present in the plant, which serve as reducing agents in the formation of metal nanoparticles [17,18,19,20]. Polyphenols have anticancer properties, as they can suppress cancer cell growth and induce apoptosis through various signaling pathways [19,20]. Green-synthesized ZnO nanoparticles have been effectively used in clinical and preclinical research for targeted medication delivery and have been proven to be safe when administered orally [21].

Hormone-related cancers, such as prostate cancer (PCa) and thyroid cancer (TC), have been reported to have excessive reactive oxygen species (ROS) production from toxic cholesterol contributing to cancer progression [22,23,24]. Therefore, our study proposes the use of green tea extract (*Camellia sinensis*) for synthesizing ZnO NPs/SNPs, leveraging its dual role as a reducing and stabilizing agent in a one-pot template synthesis. Moreover, we synthesized fluorescent-conjugated hierarchical structures of ZnO nanoparticles for future target drug delivery and diagnosis. The anti-cancer effects of these components were further evaluated in vitro. Additionally, we also elucidated the mechanism of polyphenols targeting ROS induced by cholesterol in hormone-related cancers with the histopathological validation of safe administration in vivo.

## 2. Materials and Methods

Materials: Green tea (GT) (*Camellia sinensis*) extract powder was purchased from AP Operations Co., Ltd. (Chonburi, Thailand). Zinc acetate dihydrate was acquired from Merck (Darmstadt, Germany), and distilled water was used throughout the experiments.

Preparation of total phenols (TP) as reducing agent and stabilizing agent: GT powder was irradiated with γ-rays using 60Co gamma rays from Gamma Cell 220 irradiator (Atomic Energy of Canada Limited, Ottawa, ON, Canada) 11 kGy/h at 10 kGy under ambient temperature in air. After irradiation, GT-10 powder was used to produce polyphenol. The TP extraction process was conducted as follows: 60 mg of GT-10 powder from irradiated GT was dissolved in 10 mL of DI water and mixed with an equal volume of 50% ethanol (*w*/*v*). The solution was incubated at room temperature for 25 min and centrifuged at 13,000 rpm and 4 °C for 10 min. The supernatant was discarded, and the TP pellet was dried at 60 °C for 2 days. After extraction, the obtained TP was characterized and confirmed using UV-vis spectroscopy (Agilent Technologies, Santa Clara, CA, USA).

Preparation of ZnO NPs and ZnO HSs for cellular phenotypes experiment: Total phenol extracts and Zn acetate synthesized from previous methods were separately dissolved in 3 mL of deionized water (vortexed vigorously). Then, TP extract solution and Zn acetate were mixed gently in a light-protected container (vortexed vigorously for 10 s). The mixture was then incubated at room temperature (in a dark environment) for 12–15 h to form a ZnO NPs solution. After the incubation, the dark brown precipitate revealed the formation of ZnO NPs. The mixture was then stirred for 30 min or sonicated at 25 °C for 15 min for equal distribution of the particles. Then, both solutions of ZnO NPs and dried ZnO HSs were diluted to various concentrations before the subsequent cellular experiment.

In vitro validation: Four human prostate cancer (PCa) cell lines (PC-3, DU-145, LNCaP, 22RV1), Three human thyroid cancer cell lines (TPC-1, BCPAP, 8305C) and Benign Prostatic Hyperplasia (BPH-1) were purchased from Cell Bank of Chinese Academy of Sciences (Shanghai, China) and were cultured in Roswell Park Memorial Institute medium (RPMI 1640) (Gibco, Grand Island, NY, USA) with 10% heat-inactivated fetal bovine serum (FBS) and 1% antibiotic-antimycotic (ThermoFisher Scientific, Waltham, MA, USA). All cell lines were incubated at 37 °C with a 5% CO_2_ supply. Methyl thiazolyl tetrazolium (MTT) assay (Roche, Basel, Switzerland) was performed to evaluate cell proliferation and identify half-maximal inhibitory concentration (IC50) for NPs and SNPs in each cell line (Supplementary Methods). Subsequent evaluations were conducted to evaluate the treatment efficacy of ZnO NPs and SNPs (Supplementary Information): (1) Transwell migration and invasion assay; (2) cell cycle analysis were performed using Cell Analyzer Agilent NovoCyte Quanteon; (3) Caspase-3 activity was measured using a Caspase-3 Activity Colorimetric Assay Kit (Elabscience, Wuhan, China); (4) total cholesterol was determined using Total Cholesterol Assay Kit (Yeasen Biotechnology, Shanghai, China); (5) 3D spheroid size and morphology were generated via Aggrewell400 24 well plates (Stemcell Technologies, Vancouver, BC, Canada) in accordance with normal cell culture protocols; and (6) the levels of intracellular ROS were determined using a Reactive Oxygen Species Assay Kit (Beyotime, Shanghai, China).

Cholesterol-mediated ROS production Pathway Evaluation: The cells were washed with PBS twice and then changed to RPMI 1640 medium containing 50 μg/mL water-soluble cholesterol (CHO) (Sigma-Aldrich, St. Louis, MO, USA) with or without ZnO HSs treatment for 2 h. Then the cells were collected to perform the subsequent experiments. The levels of intracellular ROS were determined using a Reactive Oxygen Species Assay Kit (Beyotime, Shanghai, China). Cells were incubated with 10 μM H2DCFDA (Beyotime, Shanghai, China). in the dark for 20–30 min and then washed twice with deionized water 3 times. The fluorescence intensity was read at 525 nm and 580 nm, respectively, using a Microplate Reader (BMG CLARIOstar Plus, Ortenberg, Germany). The data were analyzed as relative fold change in ROS.

In vivo toxicity evaluation. Male BALB/c nude mice (4–6 weeks) were maintained in a specific-pathogen-free (SPF) environment and had access to food and water. The BALB/c nude mice were obtained from Zhuhai Bestest Biotechnology Co., Ltd, Zhuhai, China. The mice were raised and maintained at Shenzhen Huarui Model Biotechnology Co., Ltd, Shenzhen, China. The experimental protocol was approved by the Institutional Animal Care and Use Committee (IACUC) of Shenzhen Huarui Model Biotechnology Co., Ltd. with HKU-SZ Hospital (China); No. APS-260224-0038-001 (Approval date: 15 April 2026) in accordance with GB/T 35892-2018; Laboratory animal—Guideline for ethical review of animal welfare. It was published by the Standardization Administration of the People’s Republic of China (SAC) in Beijing, China, in 2018. After 14 days of ZnO NPs and ZnO HSs treatment (0.05 mg/kg daily), the mice were euthanized. Major organs (liver, kidneys, spleen, lungs) were collected, Formalin-Fixed, and Paraffin-Embedded (FFPE) to perform Hematoxylin and Eosin Staining (H&E) for histopathological observation for toxicity.

Statistical analyses. GraphPad software 10 (GraphPad Software, CA, USA) was used as a tool to perform analyses from at least three independent experiments with the mean ± standard deviation (SD). Kruskal–Wallis statistic (non-parametric test) was used to analyze differences in each experimental group and *p* value < 0.05 was considered statistically significant (**** *p* < 0.0001; *** *p* < 0.001; ** *p* < 0.01; * *p* < 0.05; ns, no significant difference).

## 3. Results

### Synthesis and Characterization of ZnO NPs and ZnO HSs

**Figure 1:**
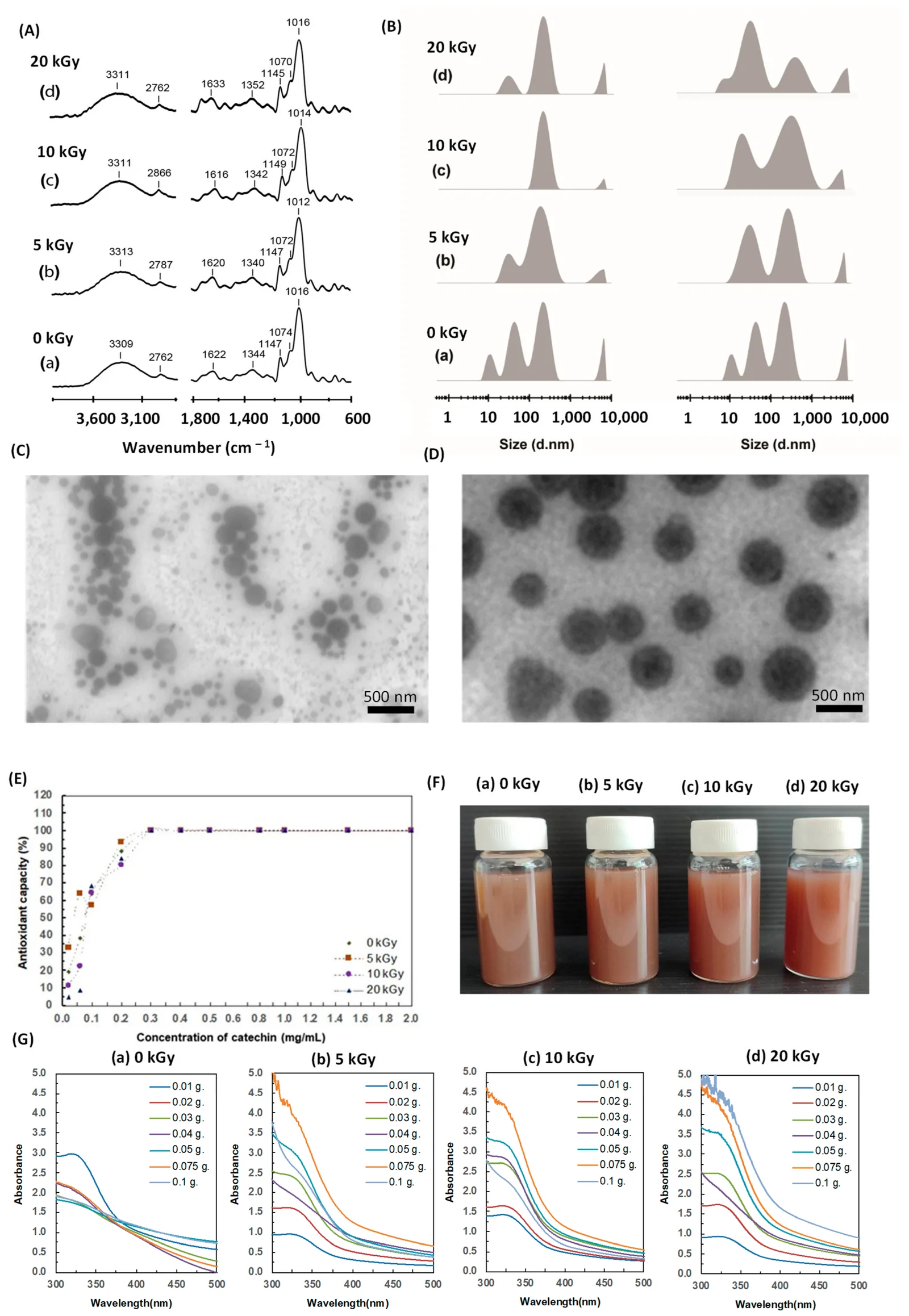
FTIR spectrum (**A**) and hydrodynamic size (**B**) of irradiated green tea using gamma rays at (**a**) 0, (**b**) 5, (**c**) 10 and (**d**) 20 kGy. TEM images of irradiated green tea at (**C**) 0 and (**D**) 10 kGy. Antioxidant activity of GT irradiated using gamma-rays at 0, 5, 10 and 20 kGy (**E**). Physical appearance (**a**) 0, (**b**) 5, (**c**) 10 and (**d**) 20 kGy (**F**) and UV-vis spectra (**a**) 0, (**b**) 5, (**c**) 10 and (**d**) 20 kGy (**G**) presenting the ZnO NPs formation in different concentrations of GT (0.01–0.1 g/mL) at (**a**) 0, (**b**) 5, (**c**) 10 and (**d**) 20 kGy with concentrations of Zn^2+^ precursor (**a**,**c**) 0.2 M and (**b**,**d**) 0.3 M.

The synthesis and characterization of irradiated green tea (GT) using different kGy of gamma rays by FTIR spectrum and hydrodynamic size showed the decreasing of size distribution after GT was irradiated, and it shows the homogenous particle size at 550 nm in a 10 kGy gamma ray condition (Figure 1A,B). TEM images illustrated the morphology of irradiated green tea GT at 0 and 10 kGy (Figure 1C,D). The GT irradiated at 10 kGy has an almost similar spherical shape, around 200–400 nm. Antioxidant activity of GT irradiated using gamma-rays at 0, 5, 10 and 20 kGy is displayed in Figure 1E. Figure 1F,G shows physical appearance and UV-vis spectra presenting the ZnO NPs formation in different concentrations of GT (0.01–0.1 g/mL) at (a,b) 0 kGy, (c,d) 10 kGy with concentrations of Zn^2+^ precursor (a,c) 0.2 M and (b,d) 0.3 M.

**Figure 2:**
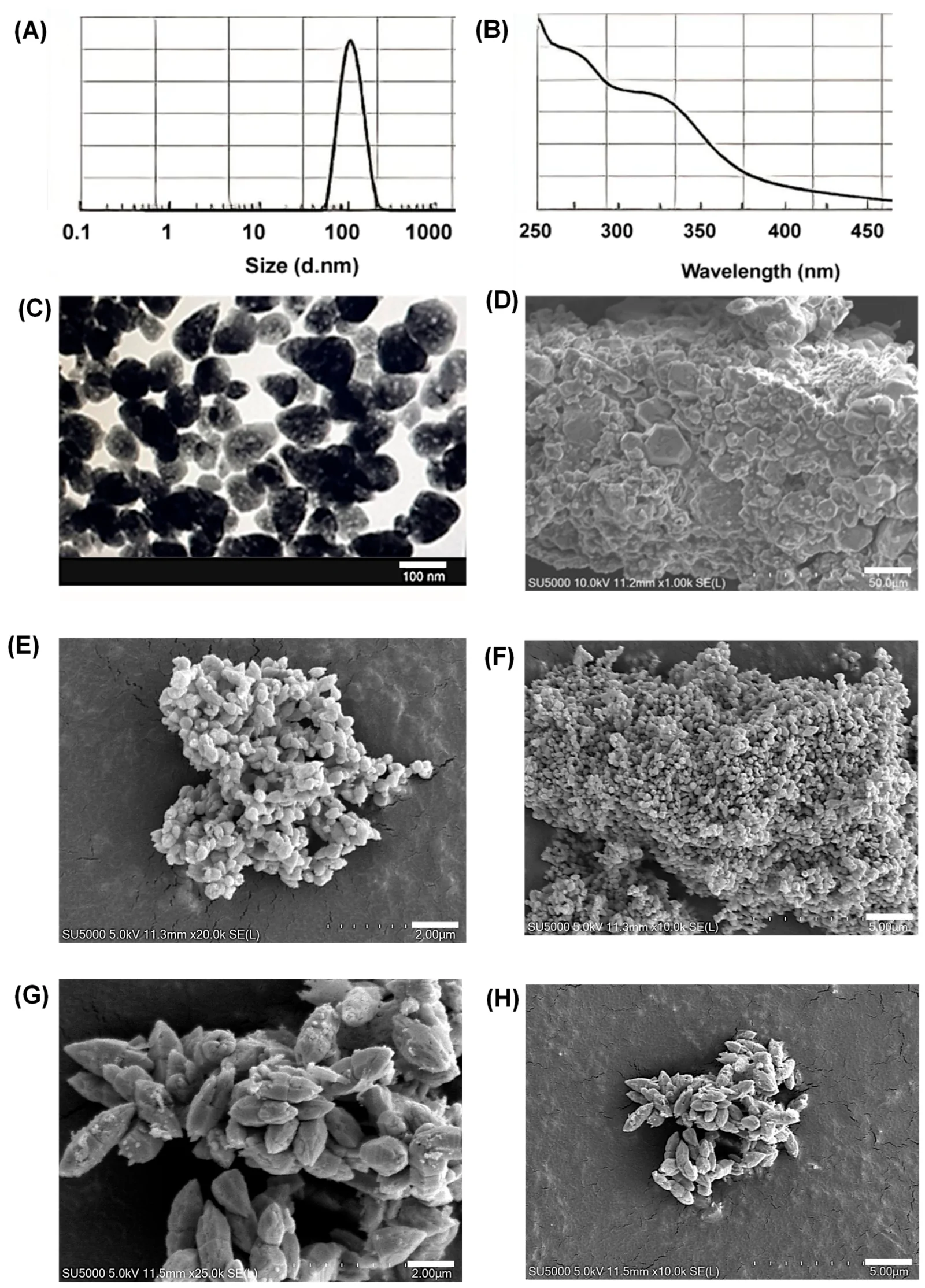
Characterization of ZnO NPs and Synthesis of ZnO HSs. The hydrodynamic diameter of ZnO NPs is 109 ± 26 nm with a zeta potential of −0.4 ± 0.1 mV (**A**). The UV-Vis result revealed the formation of the nanoparticles (**B**). TEM unveiled the structure and characterization of ZnO NPs synthesis with a size of 53 ± 12 nm (**C**). SEM images revealed the structure of ZnO NPs (Scale bar = 50 μm.) (**D**). SEM images illustrated the structure of ZnO HSs after 5 h of synthesis at 2 μm and 5 μm (**E**). SEM images illustrated the structure of ZnO HSs after 5 h of synthesis at 5 μm (**F**). SEM images illustrated the structure of ZnO HSs after 12 h of synthesis at 2 μm (**G**). SEM images showed the hierarchical structure of ZnO HSs after 12 h of synthesis at 5 μm (**H**).

The synthesis and characterization of ZnO NPs/SNPs were performed using UV-vis spectrophotometry, Dynamic Light Scattering (DLS), FTIR, XRD and TEM. hydrodynamic diameter of ZnO NPs from Dynamic Light Scattering (DLS) showed a peak at 109 ± 26 nm (Figure 2A). The absorption spectrum of the samples was recorded in the range of 280–350 nm, showing absorbance peaks at 280 and 340 nm, which correspond to the characteristic bands of extracted ZnO NPs, respectively (Figure 2B). TEM and SEM images showed the spherical morphology of green-synthesized ZnO NPs at 100 nm and 50 μm respectively (Figure 2C,D). As shown in Figure 2E,F, the spherical morphology of ZnO HSs was characterized by SEM, showing the detailed patterns of ZnO HSs at 5 h (2 μm and 5 μm respectively). Figure 2G,H shows the hierarchical structure morphology of ZnO HSs after 12 h of synthesis (2 μm and 5 μm respectively).

**Figure 3:**
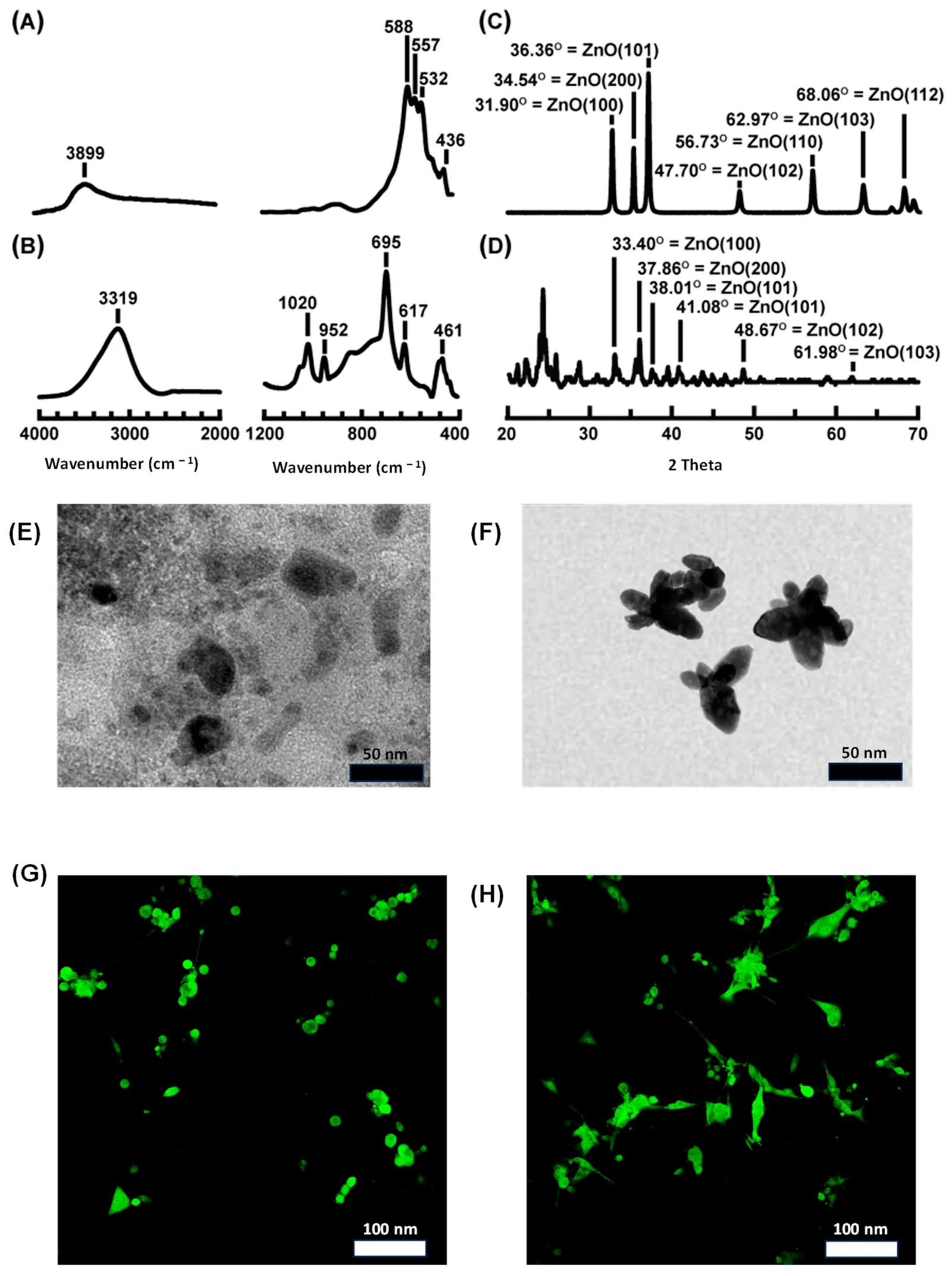
Characterization of ZnO NPs and ZnO HSs. FTIR spectra of ZnO NPs characterization depicting chemical bonds and functional groups in ZnO NPs and ZnO HSs respectively (**A**,**B**). (**C**,**D**) X-ray diffraction (XRD) illustrating patterns of ZnO NPs and ZnO HSs (**A**,**B**). Representative TEM images revealing morphology of ZnO NPs and ZnO HSs (Scale bar = 50 nm and 500 nm) (**E**,**F**). Representative confocal images of FITC-conjugated ZnO NPs and ZnO HSs (Scale bar = 100 nm) (**G**,**H**).

The FTIR spectra showed distinct absorption bands for spherical ZnO NPs (Figure 3A) and ZnO HSs (Figure 3B). The FTIR spectra of ZnO NPs exhibit a characteristic Zn–O bonding peak at 588 cm^−1^, and strong and broad peaks corresponding to O–H bond stretching were observed, with a sharp absorption peak at 3899 cm^−1^ attributed to the alcohol group. Moreover, the FTIR spectrum of ZnO HSs showed a peak at 3319 cm^−1^, indicating O–H bond stretching. This difference from ZnO NPs may suggest variations in surface hydroxylation or hydration, potentially affecting dispersion, stability, and reactivity. In addition, the FTIR spectrum of ZnO HSs exhibited the presence or absence of peaks around 1600–1750 cm^−1^ (C=O stretch) and 2800–3000 cm^−1^ (C–H stretch), suggesting possible functionalization with organic groups to enhance properties including hydrophobicity and biocompatibility.

XRD patterns for ZnO NPs (Figure 3C) and ZnO HSs (Figure 3D), labeled with Miller indices (hkl) for the hexagonal wurtzite phase, demonstrated characteristic diffraction peaks corresponding to ZnO crystal planes. Figure 3C, ZnO NPs shows diffraction peaks at 31.90°, 34.54°, 36.36°and 47.70° correspond to (100), (200), (101) and (102) of crystal planes of ZnO NPs (JCPDS Card No. 36-1451), whereas ZnO HSs showed sharper and more intense peaks at 33.40°, 37.86° 38.01° and 48.67° in (100), (200), (101) and (102) crystal planes, indicating higher crystallinity (Figure 3D).

The results indicated that the broader peaks in ZnO NPs suggested smaller crystallite sizes or possible amorphous content. TEM images revealed significant differences in the structural morphology, with ZnO NPs (Figure 3E) appearing nearly spherical, indicative of uniform growth, while ZnO HSs (Figure 3F) exhibited hierarchical structures, suggesting anisotropic growth. Figure 3G,H, and Supplementary Figures S1 and S2 revealed the successful FIT-C conjugation of both ZnO NPs and ZnO HSs.

The anticancer activity of ZnO NPs was dose-dependent across PCa and TC cell lines (Supplementary Figure S3). After 48 h of treatment, IC50 values for ZnO NPs in PC3, DU-145, 22RV1, LNCaP, 8305C, BCPAP, and TPC-1 were determined (26.80~35.22 µg/mL) (Supplementary Figure S3A–G), with BPH-1 showing an IC50 of 116.3 µg/mL (Supplementary Figure S3H), indicating safety for the normal cell line. ZnO HSs exhibited higher potency than ZnO NPs, with lower IC50 values across cell lines (13.04 µg/mL in PC3, 21.37 µg/mL in DU-145, 26.73 in 22RV1, 34.18 µg/mL in LNCaP, 25.33 µg/mL in BCPAP, 31.08 µg/mL in TPC-1 and 18.56 µg/mL in 8305C (Supplementary Figure S3A–G). Lowest toxicity was also observed in BPH-1 with an IC50 of 79.28 µg/mL. Supplementary Figure S4 showed successful cellular uptake and location of ZnO NPs and ZnO HSs in PC3 and 8305C (representative cell lines from PCa and TC).

**Figure 4:**
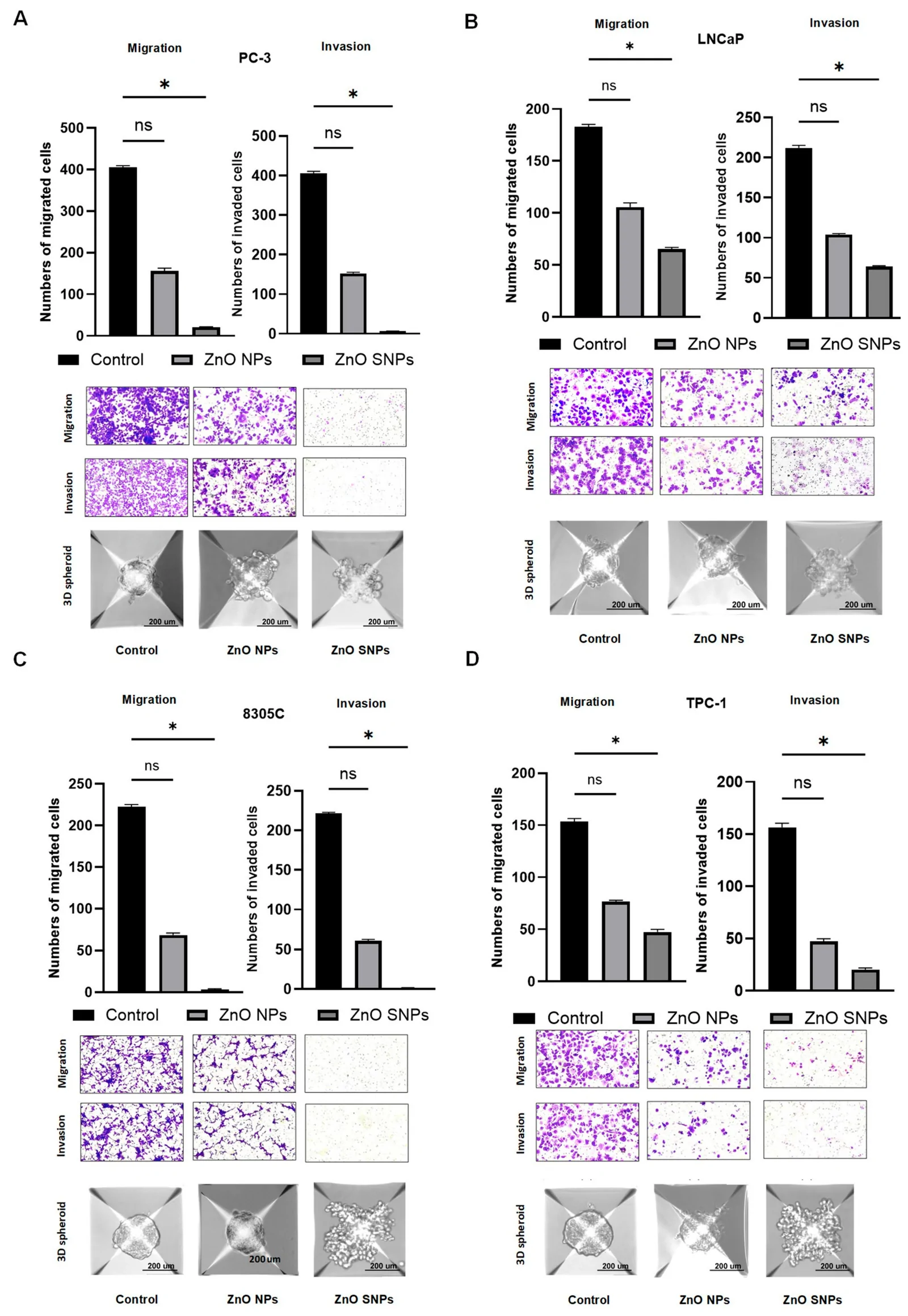
Evaluation of metastatic ability after ZnO NPs and ZnO HSs treatment: (**A**–**D**) The representative images and analyses of Transwell migration assay and invasion assay showing treatment of ZnO NPs and ZnO HSs inhibited the migration and invasion of PCa (PC3 and LNCaP) and TC cells (8305C and TPC-1) (magnification: 100×). N amount of 0.2% Crystal Violet was used to stain cells, indicating the migrated and invaded cells. The morphology of 3D PCa (PC3 and LNCaP) cells and TC (8305C and TPC-1) spheroids using Aggrewell400 after 48 h of ZnO NPs and ZnO HSs treatment. The scale bar of the images is 200 µm. In all panels, error bars represent mean ± s.d. (*n* = 3). Non-parametric tests were used to analyze the data (ns = not significant and * = *p* < 0.05).

**Figure 5:**
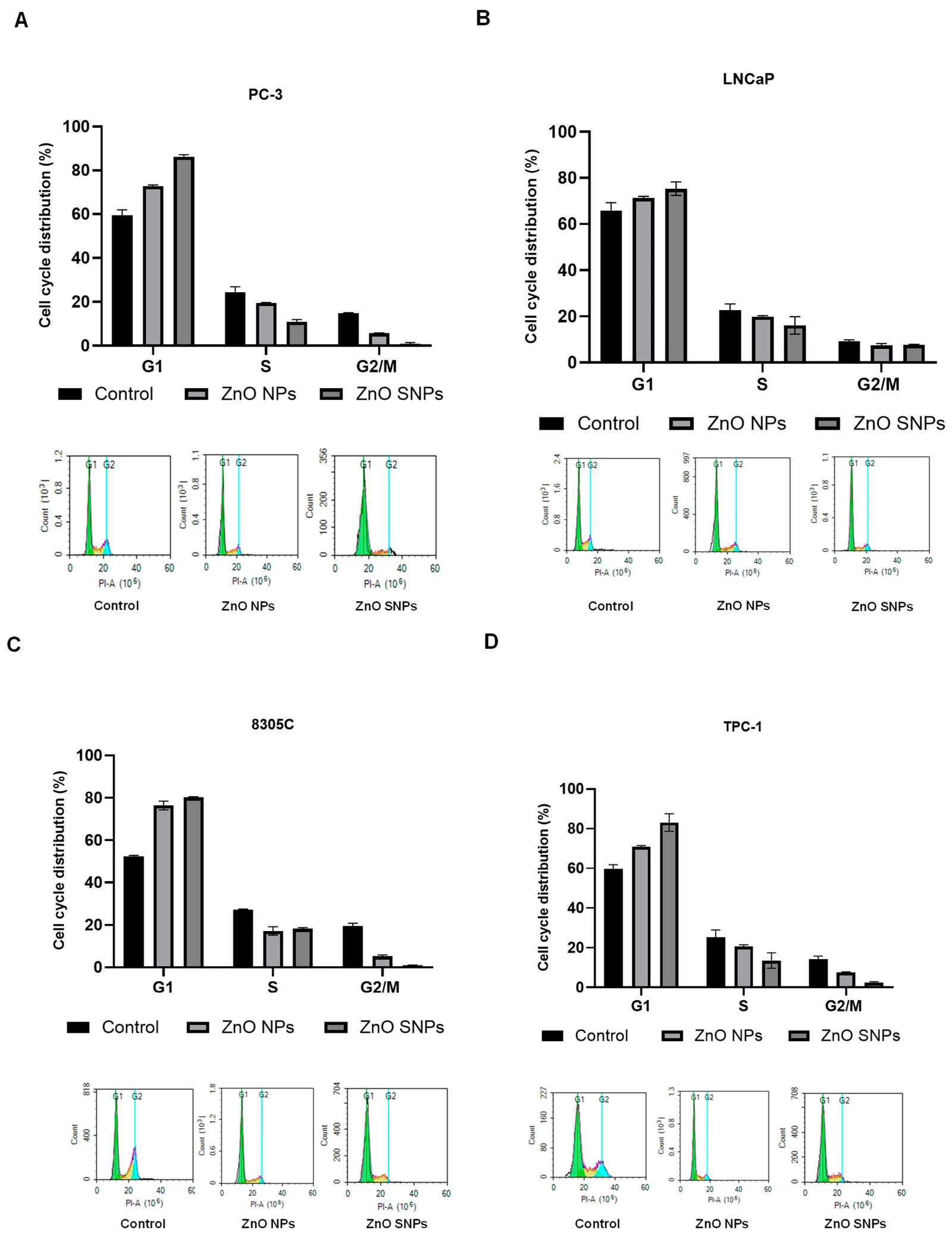
Cell cycle analysis of PCa and TC cell lines. (**A**–**D**) The representative images and analyses of cell cycle analysis by flow cytometry of PCa (PC3 and LNCaP) and TC cells (8305C and TPC-1) with ZnO NPs and ZnO HSs treatment for 48 h. In all panels, error bars represent mean ± s.d. (*n* = 3). Non-parametric tests were used to analyze the data.

Migration and invasion assays demonstrated a significant reduction in metastasis in treated cancer cell lines (all *p* < 0.05) (Figure 4 and Supplementary Figure S5). These effects, shown in Figure 4A,B, were more significant in cell lines with aggressive phenotypes (PC3, DU145 and 8305C) compared to non-aggressive ones (Figure 4C,D). ZnO NPs treatment also led to structural changes in 3D spheroids, with cell aggregation observed in aggressive cancer cell lines such as PC3, DU145 and 8305C. Non-aggressive ones (22RV1, LNCaP, BCPAP, TPC-1) showed less 3D dissociation after treatment (200 µm scale). Migration and invasion assays confirmed significantly fewer migrated and invaded cells after ZnO HSs treatment compared to NPs. This effect was higher in aggressive cell lines (PC3, DU145 and 8305C) than in non-aggressive ones. ZnO HSs also effectively dissociated cancer 3D spheroids. Flow cytometry showed higher G0/G1 phase arrest in aggressive cell lines treated with ZnO HSs compared to ZnO NPs (Figure 5 and Supplementary Figure S6). Flow cytometry analysis revealed an increase in the percentage of PC3, DU145, and 8305C cells in the G0/G1 phase following ZnO NP treatment, with a corresponding decrease in the G2/M phase.

**Figure 6:**
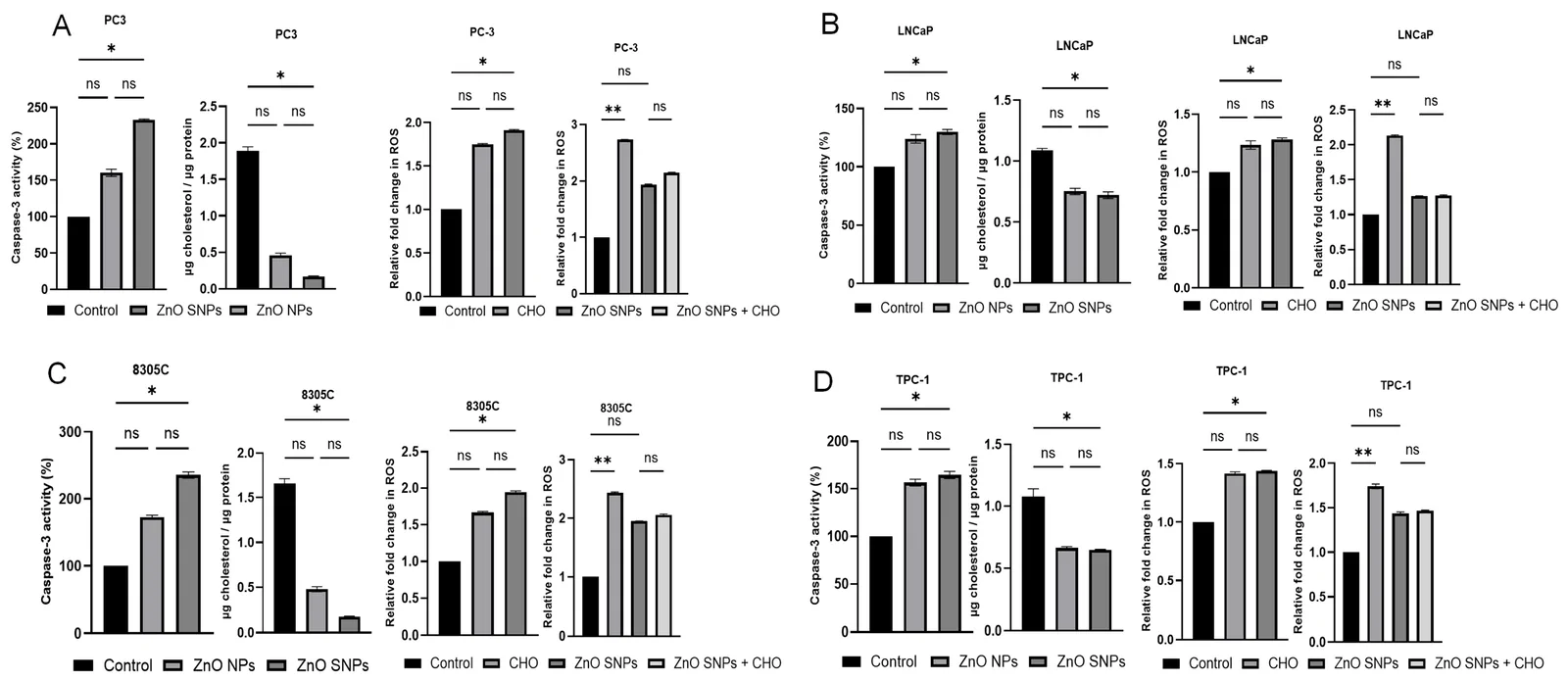
(**A**–**D**) Caspase-3 activity was detected at 405 nm after PCa (PC3 and LNCaP) and TC cells (8305C and TPC-1) treated with ZnO NPs and ZnO HSs for 48 h. The relative fold change in intracellular ROS in PCa (PC3 and LNCaP) and TC cells (8305C and TPC-1) was determined by fluorescence intensity at 525 nm and 580 nm after 2 h of CHO incubation and treatment with ZnO HSs for 48 h. Statistical values are the mean ± standard deviation (SD; vertical bars) of three independent experiments. (ns = not significant and * = *p* < 0.05, ** = *p* < 0.01).

Figure 6 and Supplementary Figure S7 illustrated apoptosis assays showed increased caspase-3 activity in all PCa and TC cell lines after ZnO NP treatment (Figure 6A–D). Total cholesterol assays indicated significantly reduced cholesterol levels post-treatment in aggressive cancer cell lines compared to non-aggressive ones (Figure 6A–D). ROS levels increased significantly in all treated PCa and TC cell lines compared to control. After treatment with CHO, ZnO HSs exhibited higher efficacy in modulating ROS levels (Figure 6A–D). Apoptotic activity was significantly higher in PC-3, DU-145, and 8305C following ZnO SNP treatment. ZnO HSs enhanced ROS levels in aggressive cell lines (all *p* < 0.05).

**Figure 7:**
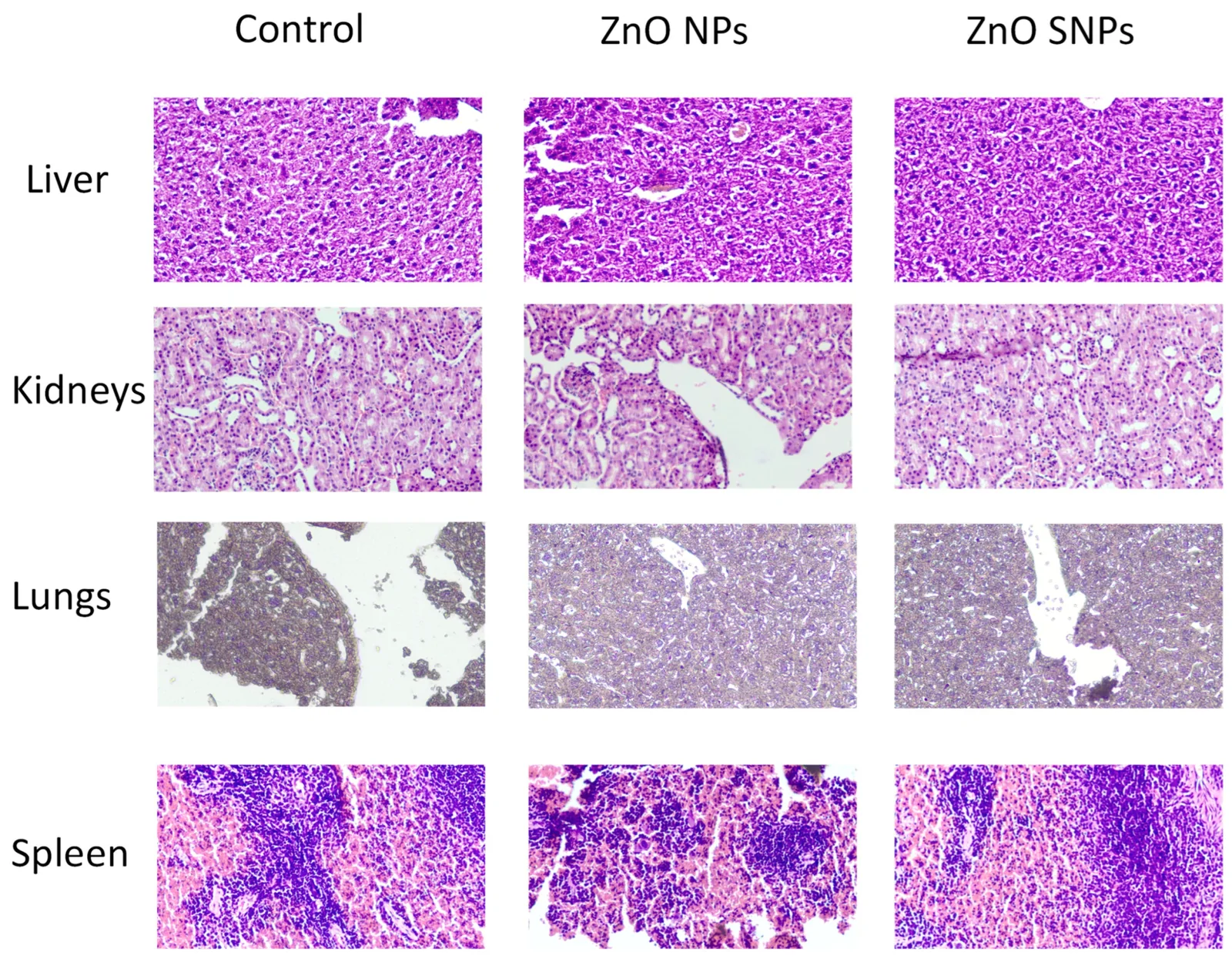
In vivo cytotoxicity of ZnO NPs and ZnO HSs after 14 days of treatment. H&E-stained histological images of tissue sections from major organs (liver, kidney, lung, and spleen).

Treatment with ZnO HSs effectively regulated cholesterol-induced ROS production in aggressive PCa and TC cell lines (PC-3, DU-145, and 8305C). Excessive ROS production was observed following cholesterol induction (*p* < 0.05) (Figure 6A–D and Supplementary Figure S8). ZnO SNP treatment resulted in significantly lower fold changes in ROS levels compared to cholesterol control in aggressive cancer cell lines (treatment vs. control: 2.14 vs. 2.73 in PC3, 2.15 vs. 2.63 in DU145, and 2.05 vs. 2.43 in 8305C). In addition, PC3 and 8305C that were supplemented with 10% Lipoprotein-Deficient Serum (LPDS) for 72 h showed lower cell viability after subsequent ZnO NPs and ZnO SNPs treatment for 48 h (Supplementary Figure S9). In non-aggressive ones and normal cell lines, there is no significant difference compared to ZnO HSs treatment, only indicating effective regulation of cholesterol-induced ROS. Figure 7 revealed no significant change between control and treatment groups in histopathology of major organs in mice, including liver, kidney, lung and spleen.

## 4. Discussion

The band appearing at 1447 cm^−1^ in the ZnO@Tetracycline hierarchical structures spectrum is assigned to C–H bending vibrations, suggesting the presence of alkane functional groups [25]. The XRD pattern of ZnO HSs in Salimi reported that the XRD peaks were obtained at 100, 002, 101, 102, 110, 103, 112, and 201 [26]. FTIR spectra of ZnO NPs synthesized with organic ligands such as citric acid or carboxylic acids are indicative of water absorption [27,28]. The enhanced crystallinity and larger crystallite size of ZnO HSs make them particularly suitable for applications demanding well-ordered ZnO structures [29,30,31]. Our study successfully synthesized and characterized ZnO NPs and ZnO HSs using green tea extract (*Camellia sinensis*), leveraging its polyphenol compounds as reducing reagents. ZnO HSs exhibit higher crystallinity compared to ZnO NPs with different crystal structures and particle sizes. Compared to previous studies, our ZnO HSs also revealed flower-like structures, indicating their penetration into blood vessels and subsequent localization within tumor cells [11,32].

The green tea-derived chemical composition of ZnO NPs likely imparts antioxidant properties, which contribute to the suppression of cancer cell proliferation and disruption of cell cycle distribution. Conversely, the hierarchical structure morphology of ZnO HSs enhances their surface area, thereby augmenting their high efficacy [31] in applications such as protein-metal nanoparticle interaction, antibacterial activity and anti-cancer activity, where increased surface interactions are advantageous [33,34].

For the first time, we successfully synthesized and characterized FIT-C ZnO and ZnO HSs, which are non-spherical ZnO NPs showing how hormone-related cancer cells internalize the medication. This sheds light on novel treatment strategies and drug delivery compared to conventional and spherical ZnO nanoparticles in previous studies since ZnO HSs possess a higher ability to penetrate 3D tumor spheroids [35,36]. Resistance to conventional anti-cancer therapies remains a significant challenge in oncology, often leading to treatment failure [37,38]. Our findings align with previous studies, demonstrating that ZnO NPs and ZnO HSs exhibit selective cytotoxicity towards aggressive hormone-related cancer cells while sparing normal cells [7,11,15,39,40,41,42]. This selective cytotoxicity underscores the potential of these nanoparticles as effective therapeutic agents. Unlike earlier studies, our research utilized a 3D spheroid model to better simulate the tumor microenvironment, enabling a more accurate assessment of the efficacy of ZnO NPs/SNPs on cell lines. Interestingly, ZnO HSs showed greater inhibitory effects on androgen receptor-negative prostate cancer cell lines (PC-3 and DU-145) and were particularly effective against anaplastic thyroid cancer cell lines (8305C). These results suggest that ZnO HSs may serve as targeted drug carriers for aggressive or drug-resistant cancer subtypes. Alterations in cholesterol metabolism significantly impact cellular membrane properties, thereby influencing nanoparticle uptake and therapeutic efficacy. Cholesterol is a critical component of the plasma membrane, contributing to membrane fluidity, organization of lipid rafts, and membrane integrity [43]. Changes in cholesterol levels can modify these properties, affecting cellular interactions with nanocarriers. Cholesterol regulates membrane fluidity by ordering the lipid bilayer [44]. Elevated cholesterol content tends to decrease membrane fluidity, making the membrane more rigid, whereas cholesterol depletion increases fluidity [45]. These changes can influence the efficiency of nanoparticle internalization; increased fluidity may facilitate endocytic processes such as clathrin-mediated endocytosis, thereby enhancing nanoparticle uptake. Alterations in cholesterol levels can disrupt or stabilize these rafts, affecting receptor clustering and localization [46]. For example, receptor-mediated endocytosis of nanoparticles often depends on raft-associated receptors; thus, cholesterol-dependent raft integrity directly influences nanoparticle internalization efficiency [47]. Cholesterol-rich membranes are susceptible to oxidative modifications, which can lead to lipid peroxidation, compromising membrane integrity and permeability [48]. Moreover, dysregulated cholesterol metabolism may influence cellular oxidative stress responses, affecting nanoparticle stability and intracellular trafficking [48]. Collectively, these membrane alterations driven by cholesterol metabolism impact nanoparticle–cell interactions. Conversely, excessive oxidative damage might reduce cellular viability or alter internalization pathways, diminishing therapeutic efficacy. Understanding how cholesterol metabolism modulates membrane properties is vital for optimizing nanoparticle-based therapeutics. Targeting cholesterol pathways or manipulating membrane composition could enhance nanocarrier uptake and improve treatment outcomes, especially in disease states characterized by cholesterol dysregulation. Previous studies have established that ZnO NPs and ZnO HSs can induce apoptosis in cancer cells through mechanism such as mitochondrial membrane potential disruption or caspase cascade activation [11,49]. Our study extends these findings by demonstrating, for the first time, that ZnO HSs can induce cancer apoptosis via cholesterol-induced ROS regulation. The use of H2DCFDA as a fluorescent probe provides a valuable measure of overall oxidative stress within cells by detecting a broad spectrum of reactive oxygen species (ROS). However, it is important to acknowledge that this method has inherent limitations, notably its inability to distinguish between different sources or types of ROS, such as mitochondrial, NADPH oxidase-derived, or other cellular sources. Consequently, while our data indicate an increase in oxidative stress under the experimental conditions, they do not elucidate the specific origins or pathways responsible for ROS generation. Recognizing this limitation is critical for accurately interpreting the mechanistic underpinnings of oxidative stress-related effects observed in our study. Future investigations employing more targeted approaches—such as mitochondrial-specific ROS probes, enzyme activity assays, or inhibitors of particular ROS-generating pathways—would provide a more detailed understanding of the sources and dynamics of oxidative stress in this context. This will enable a more precise delineation of how specific ROS sources contribute to the cellular responses observed. By modulating cholesterol levels and ROS production, ZnO HSs activate caspase-3, leading to apoptosis. This mechanism is particularly relevant given the elevated cholesterol absorption and production often observed in cancer, which contributes to aberrant metabolic processes [50]. Thus, our research highlights the dual role of ZnO HSs as both an anti-tumor agent and a translational drug carrier, offering promising strategies for targeting aggressive or drug-resistant hormone-related cancer subtypes through cholesterol-mediated ROS regulation and apoptosis.

## 5. Conclusions

In this study, we successfully synthesized and characterized ZnO NPs and Zn SNPs using both green nanotechnology and chemical methods, demonstrating their significant and robust impact on anti-tumor efficacy and metabolic activity through cholesterol-induced apoptosis mediated by ROS production. Our findings provide a proof-of-concept for a novel treatment strategy for hormone-related cancers. ZnO HSs exhibited distinct and promising effects on cytotoxicity and various in vitro phenotypes, highlighting their potential as targeted therapeutic agents. Specifically, we clarify that, under the current experimental design, it is challenging to fully decouple the effects of morphology from surface-area contributions. Future studies involving surface area normalization, detailed crystallinity analysis, and dissolution kinetics assessments are necessary to better understand the distinct roles of these factors. This acknowledgment aims to provide a transparent interpretation of our findings and to highlight the need for further investigation into morphology-dependent versus surface-reactivity-driven biological effects. Future studies should evaluate pharmacokinetics, blood circulation times, and retention profiles to better understand these variables before assessing therapeutic outcomes in vivo.

In future work, additional validation to assess the impact of such modifications on the biological activity and functionality of the nanoparticles is required. FIT-C ZnO HSs shed light on a novel treatment strategy and drug delivery comparing conventional and spherical ZnO nanoparticles since ZnO HSs possess a higher ability to penetrate 3D tumor spheroids, which can mimic the practical aspect in the clinic supporting the “from bench to bedside” principle. However, further in vivo validation is necessary to reveal no histopathological changes in major organs (liver, kidneys, spleen and lungs), emphasizing the safe administration for translational significance of both ZnO NPs and ZnO HSs in cancer metabolism and treatment, ensuring their effectiveness and safety in clinical applications.

## 6. Patents

Part of the key methodology of this research is under review by the China National Intellectual Property Administration for a patent (No. 202511110267.0).

## Data Availability

The data that support the findings of this study are available from the corresponding author upon reasonable request.

## Supplementary Materials

The following supporting information can be downloaded at: https://www.mdpi.com/article/10.3390/antiox15080935/s1. The Supplementary Figures are provided in Supplementary File. Supplementary Figure S1. Conjugation efficiency of ZnO NPs (**A**) and Conjugation efficiency of ZnO SNPs (**B**) with quantification. All data are expressed as the mean ± standard deviation (SD) from three independent experiments (*n* = 3). Non-parametric tests were used to analyze the data (ns = not significant). Supplementary Figure S2. Fluorescence intensity of ZnO NPs (**A**) and ZnO SNPs (**B**) (green) with quantification. Scale bar = 100 μm. (Quantification of Main Figure 3G,H). Supplementary Figure S3. Evaluation of ZnO NPs and ZnO HSs efficacy in vitro. (**A**–**H**) Dose response curves of PCa (PC3, DU145, 22RV1 and LNCaP), TC (8305C, BCPAP and TPC-1) cell lines and BPH-1 after treated with different concentrations of ZnO NPs and ZnO HSs for 48 hr. Cell viability of PCa and TC cell lines after ZnO NPs and ZnO HSs treatment. Data are represented as mean ± SD. Supplementary Figure S4. Cellular uptake and location of ZnO NPs and ZnO HSs (green) in PC3 (**A**) and 8305C (**B**) in nuclear region (DAPI; blue) (representative cell lines from PCa and TC). Scale bar = 100 μm. Supplementary Figure S5. Evaluation of metastatic ability after ZnO NPs and ZnO SNPs treatment. (**A**–**G**) The rep-resentative images and analyses of Transwell migration assay and invasion assay showing treat-ment of ZnO NPs and ZnO SNPs inhibited the migration and invasion of PCa (PC3, DU145, 22RV1 and LNCaP) and TC cells (8305C, BCPAP and TPC-1). (magnification: 100×). 0.2% Crystal Violet was used to stain cells indicating the migrated and invaded cells. The morphology of 3D PCa (PC3, DU145, 22RV1 and LNCaP) C cells (8305C, BCPAP and TPC-1) spheroids using Aggrewell400 after 48 hrs of ZnO NPs and ZnO SNPs treatment. The scale bar of the images is 200 µm. In all panels, error bars represent mean ± s.d. *(n* = 3). Non-parametric tests were used to analyze the data (ns = not significant and * = *p* < 0.05). Supplementary Figure S6. Cell cycle analysis of PCa and TC cell lines. (**H**–**N**) The representative images and analyses of cell cycle analysis by flow cytometry of PCa (PC3, DU145, 22RV1 and LNCaP) and TC cells (8305C, BCPAP and TPC-1) with ZnO NPs and Zno SNPs treatment for 48 h. All data are expressed as the mean ± standard deviation (SD) from three independent experiments (*n* = 3). Non-parametric tests were used to analyze the data. Supplementary Figure S7. (**A**–**H**) Caspase-3 activity was detected at 405 nm after PCa (PC3, DU145, 22RV1 and LNCaP) and TC cells (8305C, BCPAP and TPC-1) cells treated with ZnO NPs and Zno SNPs for 48 h. (**A**–**H**) Total cholesterol was analyzed after PCa (PC3, DU145, 22RV1 and LNCaP), TC cells (8305C, BCPAP and TPC-1) cells and BPH-1 treated with ZnO NPs and ZnO SNPs for 48 h. In all panels, error bars represent mean ± s.d. (*n* = 3). Non-parametric tests were used to analyze the data (ns = not significant and * = *p* < 0.05). Supplementary Figure S8. (**A**–**H**) The relative fold change of intracellular ROS in PCa cells (PC3, DU145, 22RV1 and LNCaP), TC cells (8305C, BCPAP and TPC-1) and BPH-1 were determined fluorescence intensity at 525 nm and 580 nm after treated with ZnO NPs and ZnO SNPs for 48 h. The relative fold change of intracellular ROS in PCa (PC3, DU145, 22RV1 and LNCaP) and TC cells (8305C, BCPAP and TPC-1) cells were determined fluo-rescence intensity at 525 nm and 580 nm after 2 h of CHO incubation and treated with ZnO SNPs for 48 h. In all panels, error bars represent mean ± s.d. (*n* = 3). Non-parametric tests were used to analyze the data (ns = not significant, * = *p* < 0.05 and ** = *p* < 0.01). Supplementary Figure S9. PC3 and 8305C were supplemented with 10% Lipoprotein-Deficient Serum (LPDS) in RPMI1640 for 72 hours with subsequent ZnO NPs and Zno SNPs treatment for 48 h. For cholesterol rescue, the cells were washed by PBS twice and then changed to RPMI 1640 medium containing 50 μg/mL water-soluble cholesterol (CHO) (Sigma-Aldrich, USA) with or without ZnO SNPs treatment for 2 h. Then the cells were collected to perform the MTT assay for cell viability. In all panels, error bars represent mean ± s.d. (*n* = 3). Non-parametric tests were used to analyze the data (**** = *p* < 0.0001).

## Abbreviations

PCa, prostate cancer; TC, thyroid cancer; ZnO NPs, zinc oxide nanoparticles; ZnO HSs, zinc oxide hierarchical structures; ROS, reactive oxygen species.

## References

[1] Delalat R., Sadat Shandiz S.A., Pakpour B. (2022). Antineoplastic effectiveness of silver nanoparticles synthesized from *Onopordum acanthium* L. extract (AgNPs-OAL) toward MDA-MB231 breast cancer cells. Mol. Biol. Rep..

[2] Math H.H., Shashiraj K.N., Kumar R.S., Rudrappa M., Bhat M.P., Basavarajappa D.S., Almansour A.I., Perumal K., Nayaka S. (2023). Investigation of In Vitro Anticancer and Apoptotic Potential of Biofabricated Silver Nanoparticles from *Cardamine hirsuta* (L.) Leaf Extract against Caco-2 Cell Line. Inorganics.

[3] Nandhini J., Karthikeyan E., Rajeshkumar S. (2024). Green synthesis of zinc oxide nanoparticles: Eco-friendly advancements for biomedical marvels. Resour. Chem. Mater..

[4] Alrajhi A.H., Ahmed N.M., Shanker U., Hussain C.M., Rani M. (2022). Green Synthesis of Zinc Oxide Nanoparticles Using Salvia officinalis Extract. Handbook of Green and Sustainable Nanotechnology: Fundamentals, Developments and Applications.

[5] Ezhuthupurakkal P.B., Ariraman S., Arumugam S., Subramaniyan N., Muthuvel S.K., Kumpati P., Rajamani B., Chinnasamy T. (2018). Anticancer potential of ZnO nanoparticle-ferulic acid conjugate on Huh-7 and HepG2 cells and diethyl nitrosamine induced hepatocellular cancer on Wistar albino rat. Nanomed. Nanotechnol. Biol. Med..

[6] Ostrovsky S., Kazimirsky G., Gedanken A., Brodie C. (2009). Selective cytotoxic effect of ZnO nanoparticles on glioma cells. Nano Res..

[7] Perumal P., Sathakkathulla N.A., Kumaran K., Ravikumar R., Selvaraj J.J., Nagendran V., Gurusamy M., Shaik N., Gnanavadivel Prabhakaran S., Suruli Palanichamy V. (2024). Green synthesis of zinc oxide nanoparticles using aqueous extract of shilajit and their anticancer activity against HeLa cells. Sci. Rep..

[8] Mongy Y., Shalaby T. (2024). Green synthesis of zinc oxide nanoparticles using *Rhus coriaria* extract and their anticancer activity against triple-negative breast cancer cells. Sci. Rep..

[9] Shandiz S.A.S., Sharifian F., Behboodi S., Ghodratpour F., Baghbani-Arani F. (2021). Evaluation of Metastasis Suppressor Genes Expression and In Vitro Anti-Cancer Effects of Zinc Oxide Nanoparticles in Human Breast Cancer Cell Lines MCF-7 and T47D. Avicenna J. Med. Biotechnol..

[10] Nsairat H., Abdulsahib W.K., Jyothi S.R., Nayak P.P., Chauhan A.S., Singla S., Sead F.F., Polatova D. (2026). Evaluating the mechanisms and therapeutic potential of ZnO nanoparticles as selective anticancer agents for lung malignancies. Discov. Nano.

[11] Dang Z., Sun J., Fan J., Li J., Li X., Chen T. (2021). Zinc oxide spiky nanoparticles: A promising nanomaterial for killing tumor cells. Mater. Sci. Eng. C.

[12] Hussain Z., Gou S., Liu X., Li M., Zhang H., Ren S., Han R., Liu F., Zhou X., Qiu L. (2025). Enhancing tumor immunotherapy with smart nanoparticles for reprogramming macrophages and blocking the CD47/Sirpα pathway. Mater. Today Bio.

[13] Yuan H., Jiao Z., Wang T., Zhang C.-Y. (2025). Endogenously Activated Multiple DNAzyme-Encoded Nanoflowers for High-Contrast Imaging of Long Noncoding RNA and Cancer Therapy. ACS Nano.

[14] Ammar M.M., Ali R., Abd Elaziz N.A., Habib H., Abbas F.M., Yassin M.T., Maniah K., Abdelaziz R. (2025). Nanotechnology in oncology: Advances in biosynthesis, drug delivery, and theranostics. Discov. Oncol..

[15] Anjum S., Hashim M., Malik S.A., Khan M., Lorenzo J.M., Abbasi B.H., Hano C. (2021). Recent Advances in Zinc Oxide Nanoparticles (ZnO NPs) for Cancer Diagnosis, Target Drug Delivery, and Treatment. Cancers.

[16] Wang S.-W., Lee C.H., Lin M.S., Chi C.W., Chen Y.J., Wang G.S., Liao K.W., Chiu L.P., Wu S.H., Huang D.M. (2020). ZnO Nanoparticles Induced Caspase-Dependent Apoptosis in Gingival Squamous Cell Carcinoma through Mitochondrial Dysfunction and p70S6K Signaling Pathway. Int. J. Mol. Sci..

[17] Braschi A., Lo Presti R., Abrignani M.G., Abrignani V., Traina M. (2023). Effects of green tea catechins and exercise training on body composition parameters. Int. J. Food Sci. Nutr..

[18] Das P.R., Eun J.-B. (2018). A comparative study of ultra-sonication and agitation extraction techniques on bioactive metabolites of green tea extract. Food Chem..

[19] Golpour M., Ebrahimnejad P., Gatabi Z.R., Najafi A., Davoodi A., Khajavi R., Alimohammadi M., Mousavi T. (2024). Green tea-mediated synthesis of silver nanoparticles: Enhanced anti-cancer activity and reduced cytotoxicity melanoma and normal murine cell lines. Inorg. Chem. Commun..

[20] Li S., Wu H., Tollefsbol T.O. (2021). Combined Broccoli Sprouts and Green Tea Polyphenols Contribute to the Prevention of Estrogen Receptor–Negative Mammary Cancer via Cell Cycle Arrest and Inducing Apoptosis in HER2/neu Mice. J. Nutr..

[21] Jain A., Bhise K. (2025). Nano-pharmacokinetics and pharmacodynamics of green-synthesized ZnO nanoparticles: A pathway to safer therapeutic applications. Xenobiotica.

[22] Bosland M.C., Vega K., Horton L., Schlicht M.J. (2023). Hormonal and genotoxic estrogen-androgen carcinogenesis in the NBL rat prostate: A role for aromatase. Prostate.

[23] Krashin E., Piekiełko-Witkowska A., Ellis M., Ashur-Fabian O. (2019). Thyroid Hormones and Cancer: A Comprehensive Review of Preclinical and Clinical Studies. Front. Endocrinol..

[24] Song Y., Liu J., Zhao K., Gao L., Zhao J. (2021). Cholesterol-induced toxicity: An integrated view of the role of cholesterol in multiple diseases. Cell Metab..

[25] Seol Y., Ganguly K., Patil T.V., Dutta S.D., Park H., Lee J., Randhawa A., Kim H., Lim K.T. (2025). Zinc Oxide@Tetracycline Spiky Microparticles Design for Persistent Antibacterial Therapy. J. Biomed. Mater. Res. A.

[26] Salimi K. (2025). Spherical spiky ZnO/Au nanostructures for efficient photoelectrochemical water splitting. Turk. J. Chem..

[27] Abdelbaky A.S., Abd El-Mageed T.A., Babalghith A.O., Selim S., Mohamed A.M.H.A. (2022). Green Synthesis and Characterization of ZnO Nanoparticles Using *Pelargonium odoratissimum* (L.) Aqueous Leaf Extract and Their Antioxidant, Antibacterial and Anti-inflammatory Activities. Antioxidants.

[28] Samzadeh-Kermani A., Izadpanah F., Mirzaee M. (2016). The improvements in the size distribution of zinc oxide nanoparticles by the addition of a plant extract to the synthesis. Cogent Chem..

[29] Weng C.-H., Lee W.-Y., Juang Z.-Y., Leou K.-C., Tsai C.-H. (2006). Direct synthesis of suspended single-walled carbon nanotubes crossing plasma sharpened carbon nanofibre tips. Nanotechnology.

[30] Klingshirn C. (2007). ZnO: Material, Physics and Applications. ChemPhysChem.

[31] Vijayakumar S., González-Sánchez Z.I., Anbu P., Divya M., Sonamuthu J., Rajkumar M., Durán-Lara E.F., Peng Y., Yu Y., Li M. (2025). Asafoetida gum-wrapped ZnO nanoparticles: Synthesis, characterization, and anticancer potential against gastric carcinoma. Int. J. Biol. Macromol..

[32] Khursheed R., Dua K., Vishwas S., Gulati M., Jha N.K., Aldhafeeri G.M., Alanazi F.G., Goh B.H., Gupta G., Paudel K.R. (2022). Biomedical applications of metallic nanoparticles in cancer: Current status and future perspectives. Biomed. Pharmacother..

[33] Baruah S., Afre R.A., Pugliese D. (2024). Effect of Size and Morphology of Different ZnO Nanostructures on the Performance of Dye-Sensitized Solar Cells. Energies.

[34] Ludi B., Niederberger M. (2013). Zinc oxide nanoparticles: Chemical mechanisms and classical and non-classical crystallization. Dalton Trans..

[35] Al-Shehaby N., Elshoky H.A., Zidan M., Salaheldin T.A., Gaber M.H., Ali M.A., El-Sayed N.M. (2025). In vitro localization of modified zinc oxide nanoparticles showing selective anticancer effects against colorectal carcinoma using biophysical techniques. Sci. Rep..

[36] Rehman F. (2020). Synthesis and growth mechanism of ZnO nanospheres by hydrothermal process and their anticancer effect against glioblastoma multiforme. Biomed. Lett..

[37] Yehya A., Ghamlouche F., Zahwe A., Zeid Y., Wakimian K., Mukherji D., Abou-Kheir W. (2022). Drug resistance in metastatic castration-resistant prostate cancer: An update on the status quo. Cancer Drug Resist..

[38] Zhang Y., Xing Z., Liu T., Tang M., Mi L., Zhu J., Wu W., Wei T. (2022). Targeted therapy and drug resistance in thyroid cancer. Eur. J. Med. Chem..

[39] Ibraheem S., Kadhim A.A., Kadhim K.A., Kadhim I.A., Jabir M. (2022). Zinc Oxide Nanoparticles as Diagnostic Tool for Cancer Cells. Int. J. Biomater..

[40] Maheswaran H., Djearamane S., Tanislaus Antony Dhanapal A.C., Wong L.S. (2024). Cytotoxicity of green synthesized zinc oxide nanoparticles using *Musa acuminata* on Vero cells. Heliyon.

[41] Deylam M., Alizadeh E., Sarikhani M., Hejazy M., Firouzamandi M. (2021). Zinc oxide nanoparticles promote the aging process in a size-dependent manner. J. Mater. Sci. Mater. Med..

[42] Pati R., Das I., Mehta R.K., Sahu R., Sonawane A. (2016). Zinc-Oxide Nanoparticles Exhibit Genotoxic, Clastogenic, Cytotoxic and Actin Depolymerization Effects by Inducing Oxidative Stress Responses in Macrophages and Adult Mice. Toxicol. Sci..

[43] Paukner K., Králová Lesná I., Poledne R. (2022). Cholesterol in the Cell Membrane-An Emerging Player in Atherogenesis. Int. J. Mol. Sci..

[44] Khodadadi E., Khodadadi E., Chaturvedi P., Moradi M. (2025). Comprehensive Insights into the Cholesterol-Mediated Modulation of Membrane Function Through Molecular Dynamics Simulations. Membranes.

[45] Giraldo-Lorza J.M., Leidy C., Manrique-Moreno M. (2024). The Influence of Cholesterol on Membrane Targeted Bioactive Peptides: Modulating Peptide Activity Through Changes in Bilayer Biophysical Properties. Membranes.

[46] Warda M., Tekin S., Gamal M., Khafaga N., Çelebi F., Tarantino G. (2025). Lipid rafts: Novel therapeutic targets for metabolic, neurodegenerative, oncological, and cardiovascular diseases. Lipids Health Dis..

[47] Zakany F., Mándity I.M., Varga Z., Panyi G., Nagy P., Kovacs T. (2023). Effect of the Lipid Landscape on the Efficacy of Cell-Penetrating Peptides. Cells.

[48] Goicoechea L., Conde de la Rosa L., Torres S., García-Ruiz C., Fernández-Checa J.C. (2023). Mitochondrial cholesterol: Metabolism and impact on redox biology and disease. Redox Biol..

[49] Rodríguez-Barajas N., Ponce-Regalado M.D., Segura-Almendárez M.S., Rodríguez-Razon C.M., Ghotekar S., Fellah M., Pérez-Larios A. (2024). Plant-mediated synthesis and interaction of ZnO against breast and prostate cancer: Review. Results Chem..

[50] Xiao M., Xu J., Wang W., Zhang B., Liu J., Li J., Xu H., Zhao Y., Yu X., Shi S. (2023). Functional significance of cholesterol metabolism in cancer: From threat to treatment. Exp. Mol. Med..

