# Supplementary material for: Fluorescent-Conjugated ZnO Nanostructures Exhibited 3D Anti-Tumor Efficacy Against Drug-Resistant Cancers Through Cholesterol-Mediated ROS Regulation
Source: Antioxidants (Basel). 2026 Jul 28;15(8):935. doi: 10.3390/antiox15080935 (PMC13509316; doi:10.3390/antiox15080935)
Supplement: Supplementary file 1 [file antioxidants-15-00935-s001.zip › antioxidants supplementary revised.pdf]

Supplementary Materials

Fluorescent Conjugated – ZnO Nanostructures Exhibited 3D Anti-Tumor Efficacy Against Drug-Resistant Cancers Through Cholesterol-Mediated ROS Regulation

Salida Ali[1], Ontana Yotnarong[2], Yu Li[3], Ruofan Shi[1,4], Ruochen Ma[1], Chi Yao[1], Xiaohao Ruan[4], Jingyi Huang[4], Da Huang[4], Yongle Zhan[1], Theeranan Tangthong[2], Rong Na[1*]*

[1]*Division of Urology, Department of Surgery, School of Clinical Medicine, LKS Faculty of Medicine, The University of Hong Kong, Hong Kong, China*

[2]*Nuclear Technology Research and Development Center, Thailand Institute of Nuclear Technology (Public Organization), Nakhon Nayok, Thailand*

[3]*Department of Otolaryngology-Head and Neck Surgery, The Eighth Affiliated Hospital, Sun Yat-sen University, Shenzhen 518033, China*

[4]*Department of Urology, Ruijin Hospital, Shanghai Jiao Tong University School of Medicine, Shanghai, China*

Corresponding authors email: yungna@hku.hk, narong.hs@gmail.com and theeranan@tint.or.th

Supplementary Figures

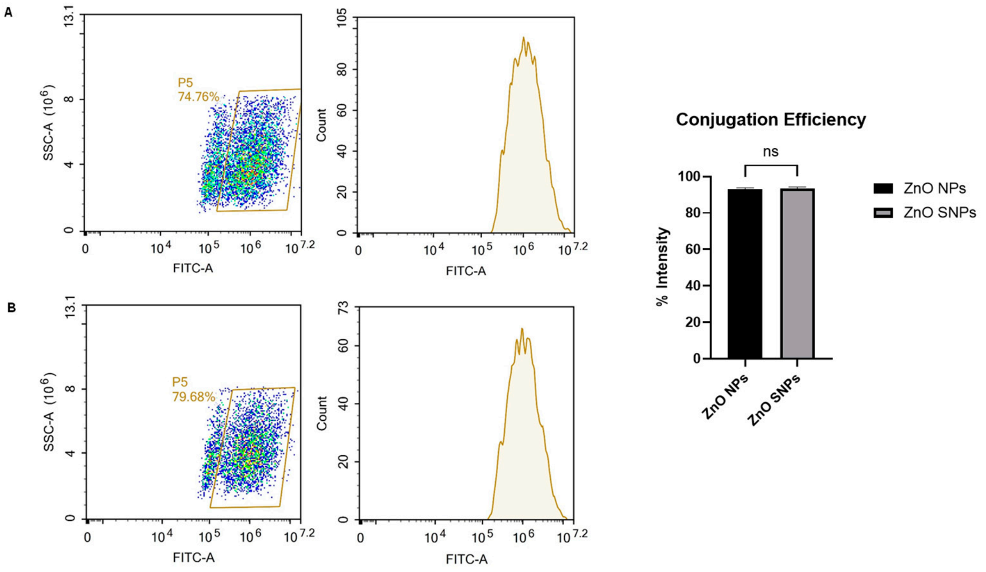

Supplementary Figure S1. Conjugation efficiency of ZnO NPs (A) and Conjugation efficiency of ZnO SNPs (B) with quantification. All data are expressed as the mean ± standard deviation (SD) from three independent experiments (n=3). Non-parametric tests were used to analyze the data (ns = not significant).

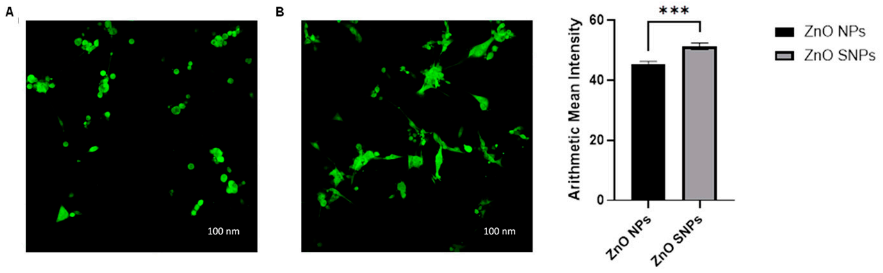

Supplementary Figure S2 Fluorescence intensity of ZnO NPs (A) and ZnO SNPs (B) (green) with quantification.

Scale bar = 100 um. (Quantification of Main Figure 3G and Figure 3H)

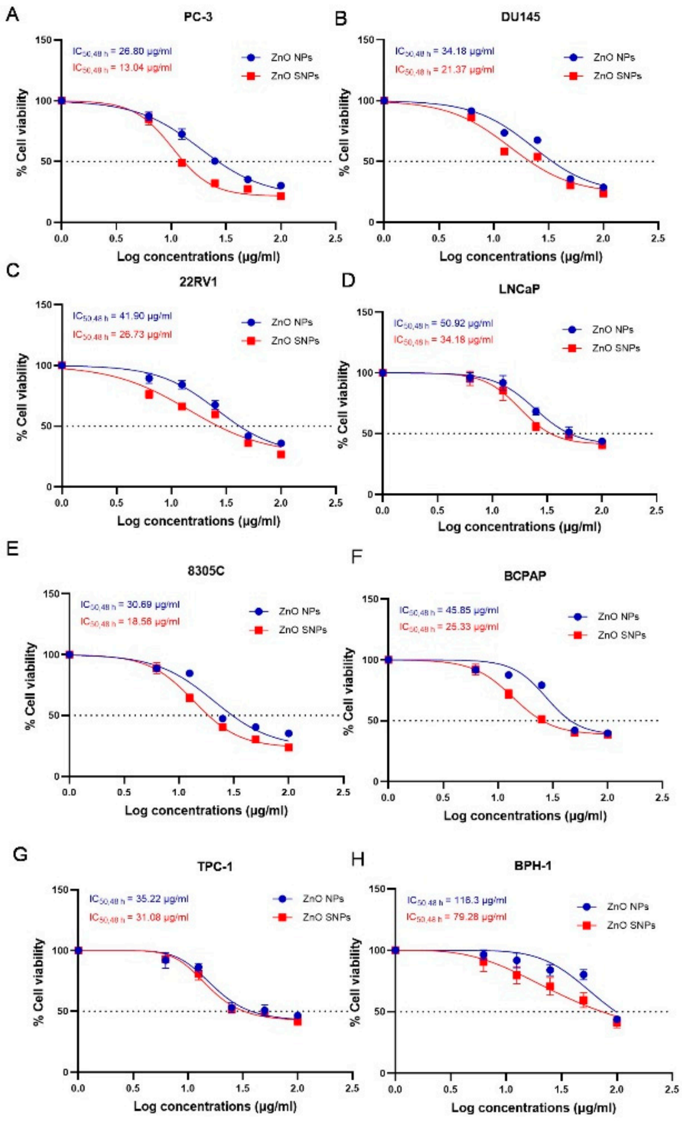

Supplementary Figure S3. Evaluation of ZnO NPs and ZnO HSs efficacy in vitro. (A-H) Dose response curves of PCa (PC3, DU145, 22RV1 and LNCaP), TC (8305C, BCPAP and TPC-1) cell lines and BPH-1 after treated with different concentrations of ZnO NPs and ZnO HSs for 48 hr. Cell viability of PCa and TC cell lines after ZnO NPs and ZnO HSs treatment. Data are represented as mean ± SD.

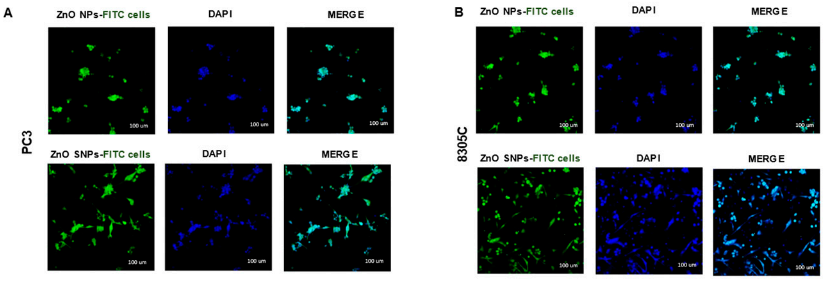

Supplementary Figure S4. Cellular uptake and location of ZnO NPs and ZnO HSs (green) in PC3 (A) and 8305C (B) in nuclear region (DAPI; blue) (representative cell lines from PCa and TC). Scale bar = 100 um.

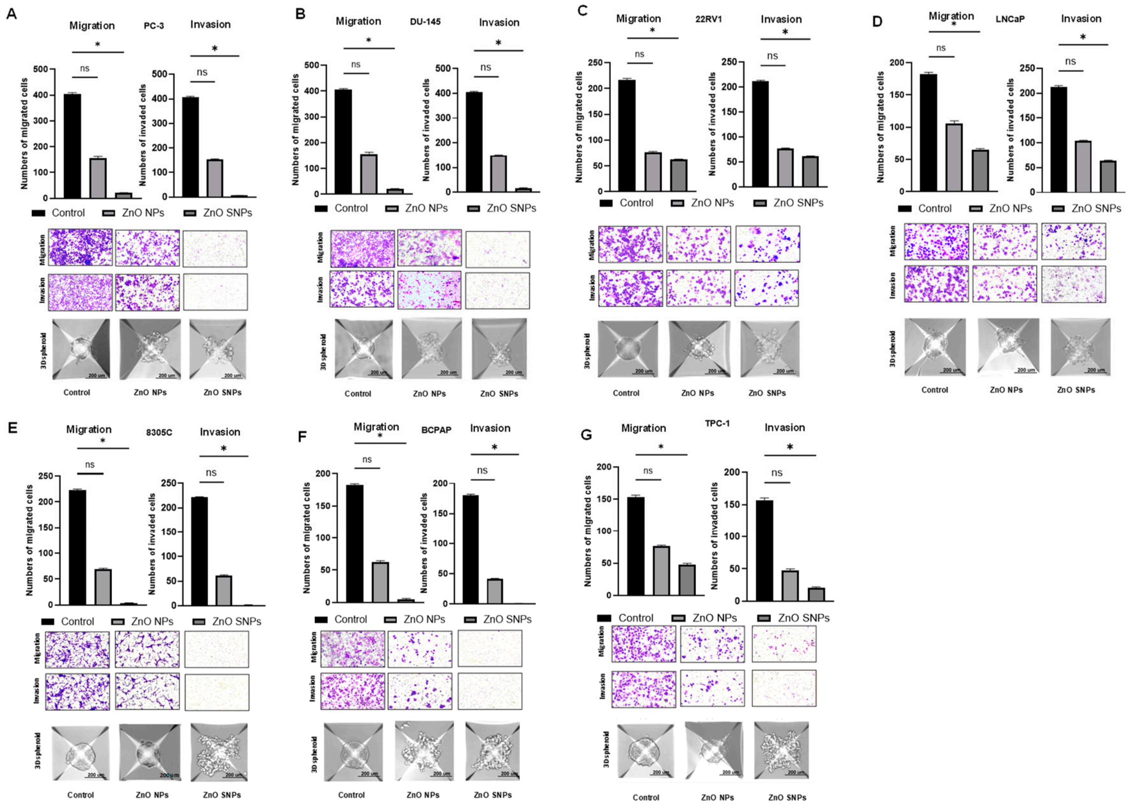

Supplementary S5. Evaluation of metastatic ability after ZnO NPs and ZnO SNPs treatment. (A-G) The representative images and analyses of Transwell migration assay and invasion assay showing treat-ment of ZnO NPs and ZnO SNPs inhibited the migration and invasion of PCa (PC3, DU145, 22RV1 and LNCaP) and TC cells (8305C, BCPAP and TPC-1). (magnification: 100x). 0.2% Crystal Violet was used to stain cells indicating the migrated and invaded cells. The morphology of 3D PCa (PC3, DU145, 22RV1 and LNCaP) C cells (8305C, BCPAP and TPC-1) spheroids using Aggrewell400 after 48 hrs of ZnO NPs and ZnO SNPs treatment. The scale bar of the images is 200 μm. In all panels, error bars represent mean ± s.d. (n = 3). Non-parametric tests were used to analyze the data (ns = not significant and * = $p < 0.05$).

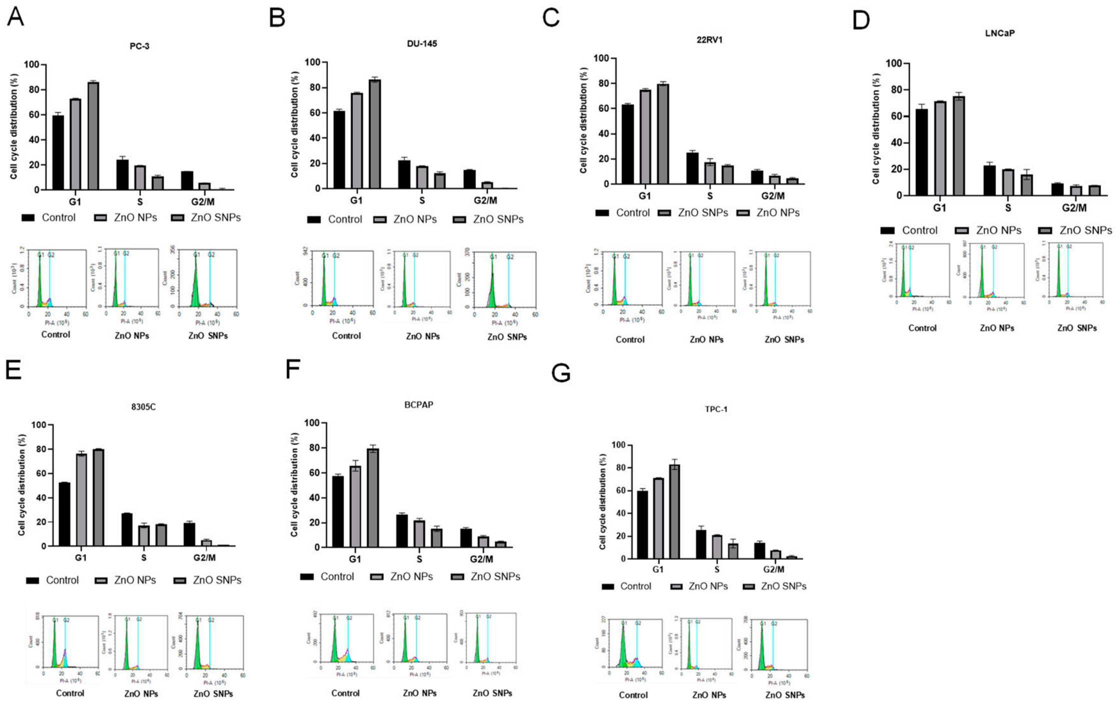

Supplementary Figure S6. Cell cycle analysis of PCa and TC cell lines. (H-N) The representative images and analyses of cell cycle analysis by flow cytometry of PCa (PC3, DU145, 22RV1 and LNCaP) and TC cells (8305C, BCPAP and TPC-1) with ZnO NPs and Zno SNPs treatment for 48 h. All data are expressed as the mean ± standard deviation (SD) from three independent experiments (n=3). Non-parametric tests were used to analyze the data.

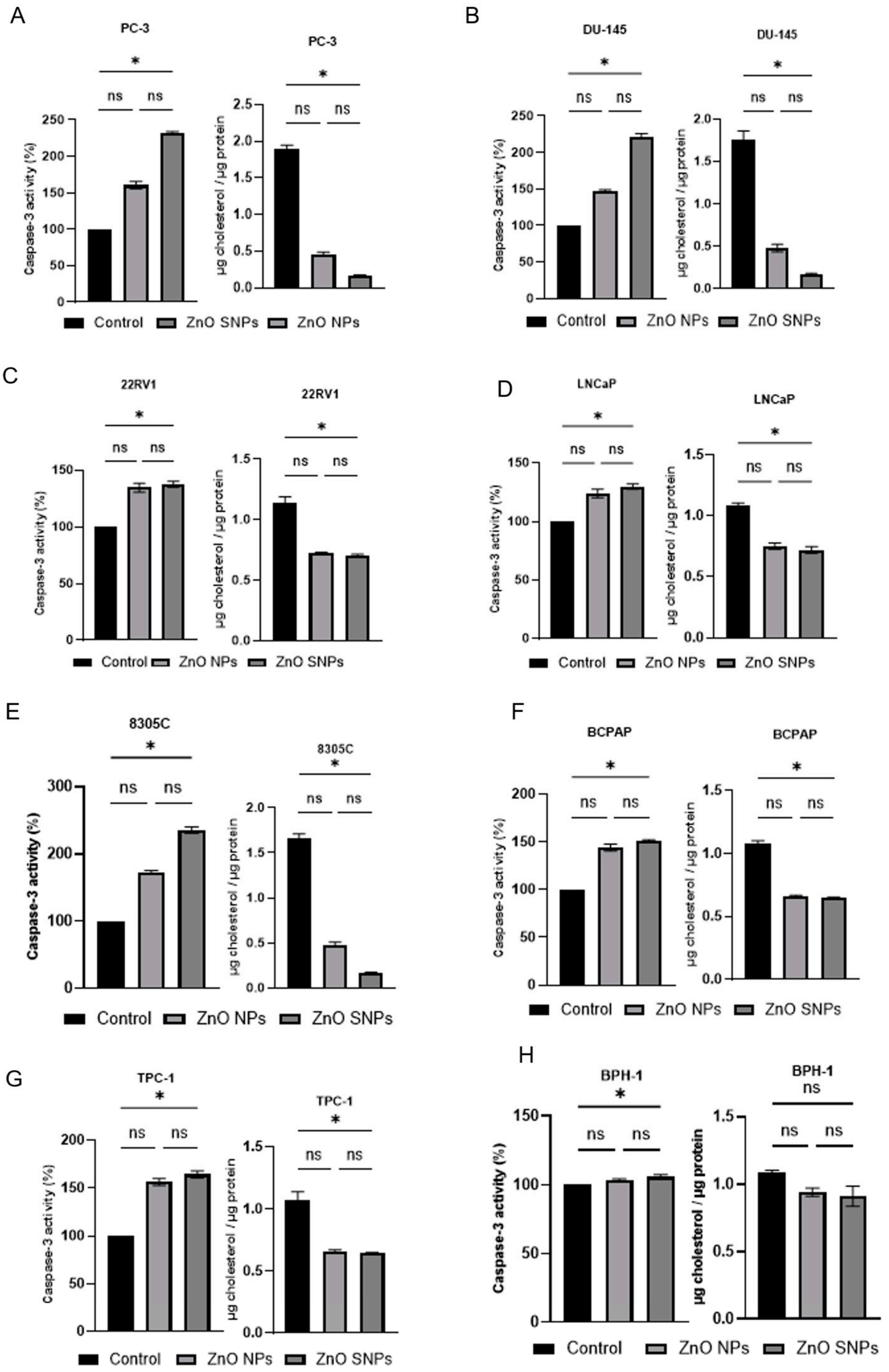
A
PC-3
PC-3
*
ns
ns
Caspase-3 activity (%)
µg cholesterol / µg protein
Control
ZnO SNPs
ZnO NPs
B
DU-145
DU-145
Caspase-3 activity (%)
µg cholesterol / µg protein
Control
ZnO NPs
ZnO SNPs
C
22RV1
22RV1
D
LNCaP
LNCaP
E
8305C
8305C
F
BCPAP
BCPAP
G
TPC-1
TPC-1
H
BPH-1
BPH-1

Supplementary Figure S7. (A-H) Caspase-3 activity was detected at 405 nm after PCa (PC3, DU145, 22RV1 and LNCaP) and TC cells (8305C, BCPAP and TPC-1) cells treated with ZnO NPs and Zno SNPs for 48 h. (A-H) Total cholesterol was analyzed after PCa (PC3, DU145, 22RV1 and LNCaP), TC cells (8305C, BCPAP and TPC-1) cells and BPH-1 treated with ZnO NPs and ZnO SNPs for 48 h. In all panels, error bars represent mean ± s.d. ($n = 3$). Non-parametric tests were used to analyze the data (ns = not significant and * = $p < 0.05$).

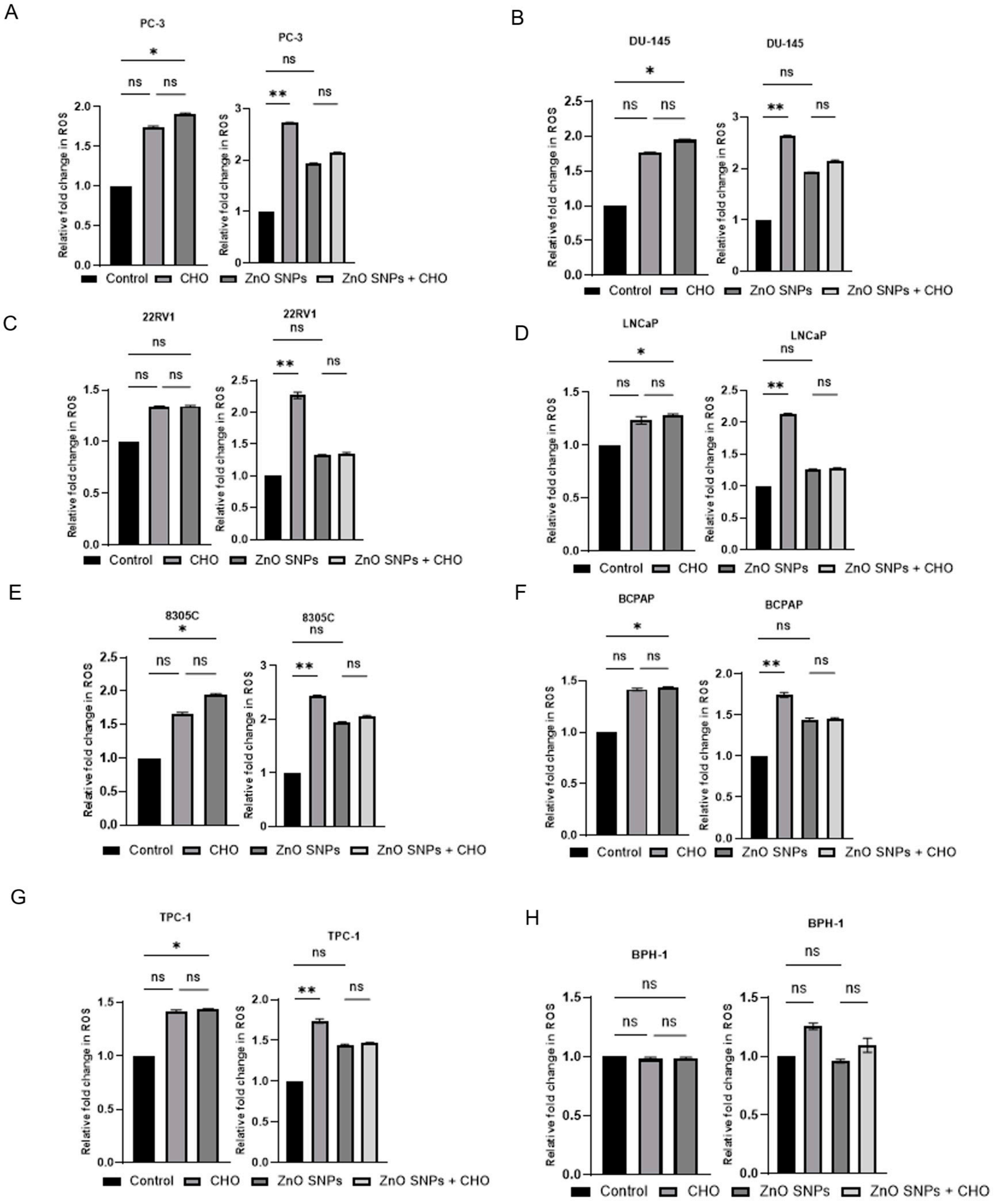
A
PC-3
PC-3
*
ns
ns
ns
**
ns
Relative fold change in ROS
2.0
1.5
1.0
0.5
0.0
3
2
1
0
Control
CHO
ZnO SNPs
ZnO SNPs + CHO
B
DU-145
DU-145
*
ns
ns
ns
**
ns
Relative fold change in ROS
2.5
2.0
1.5
1.0
0.5
0.0
3
2
1
0
Control
CHO
ZnO SNPs
ZnO SNPs + CHO
C
22RV1
22RV1
ns
ns
ns
ns
**
ns
Relative fold change in ROS
1.5
1.0
0.5
0.0
2.5
2.0
1.5
1.0
0.5
0.0
Control
CHO
ZnO SNPs
ZnO SNPs + CHO
D
LNCaP
LNCaP
*
ns
ns
ns
**
ns
Relative fold change in ROS
1.5
1.0
0.5
0.0
2.5
2.0
1.5
1.0
0.5
0.0
Control
CHO
ZnO SNPs
ZnO SNPs + CHO
E
8305C
8305C
*
ns
ns
ns
**
ns
Relative fold change in ROS
2.5
2.0
1.5
1.0
0.5
0.0
3
2
1
0
Control
CHO
ZnO SNPs
ZnO SNPs + CHO
F
BCPAP
BCPAP
*
ns
ns
ns
**
ns
Relative fold change in ROS
1.5
1.0
0.5
0.0
2.0
1.5
1.0
0.5
0.0
Control
CHO
ZnO SNPs
ZnO SNPs + CHO
G
TPC-1
TPC-1
*
ns
ns
ns
**
ns
Relative fold change in ROS
1.5
1.0
0.5
0.0
2.0
1.5
1.0
0.5
0.0
Control
CHO
ZnO SNPs
ZnO SNPs + CHO
H
BPH-1
BPH-1
ns
ns
ns
ns
ns
ns
Relative fold change in ROS
1.5
1.0
0.5
0.0
1.5
1.0
0.5
0.0
Control
CHO
ZnO SNPs
ZnO SNPs + CHO

Supplementary Figure S8. (A-H) The relative fold change of intracellular ROS in PCa cells (PC3, DU145, 22RV1 and LNCaP), TC cells (8305C, BCPAP and TPC-1) and BPH-1 were determined fluorescence intensity at 525 nm and 580 nm after treated with ZnO NPs and ZnO SNPs for 48 h. The relative fold change of intracellular ROS in PCa (PC3, DU145, 22RV1 and LNCaP) and TC cells (8305C, BCPAP and TPC-1) cells were determined fluorescence intensity at 525 nm and 580 nm after 2 h of CHO incubation and treated with ZnO SNPs for 48 h. In all panels, error bars represent mean ± s.d. (n = 3). Non-parametric tests were used to analyze the data (ns = not significant, * = $p < 0.05$ and ** = $p < 0.01$).

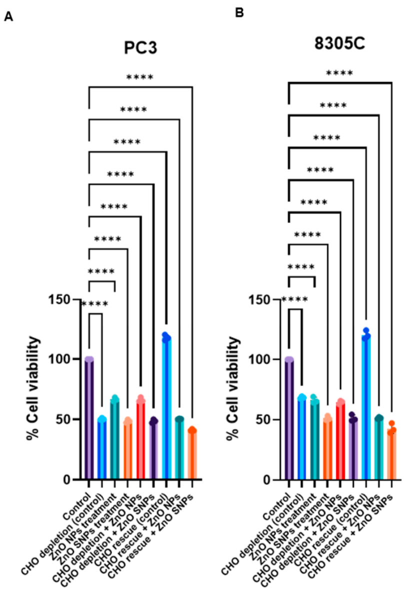

Supplementary Figure S9. PC3 and 8305C were supplemented with 10% Lipoprotein-Deficient Serum (LPDS) in RPMI1640 for 72 hours with subsequent ZnO NPs and Zno SNPs treatment for 48 h. For cholesterol rescue, the cells were washed by PBS twice and then changed to RPMI 1640 medium containing 50 μg/mL water-soluble cholesterol (CHO) (Sigma-Aldrich, USA) with or without ZnO SNPs treatment for 2 h. Then the cells were collected to perform the MTT assay for cell viability. In all panels, error bars represent mean ± s.d. (n = 3). Non-parametric tests were used to analyze the data (**** = $p < 0.0001$).
