# Supplementary material for: Fluorescent-Conjugated ZnO Nanostructures Exhibited 3D Anti-Tumor Efficacy Against Drug-Resistant Cancers Through Cholesterol-Mediated ROS Regulation
Source: Antioxidants (Basel). 2026 Jul 28;15(8):935. doi: 10.3390/antiox15080935 (PMC13509316; doi:10.3390/antiox15080935)
Supplement: Supplementary file 1 [file antioxidants-15-00935-s001.zip › Supplementary methods Antioxidants.pdf]

Supplementary methods

Fluorescent Conjugated – ZnO Nanostructures Exhibited 3D Anti-Tumor Efficacy Against Drug-Resistant Cancers Through Cholesterol-Mediated ROS Regulation

Salida Ali[1], Ontana Yotnarong[2], Yu Li[3], Ruofan Shi[1,4], Ruochen Ma[1], Chi Yao[1], Xiaohao Ruan[4], Jingyi Huang[4], Da Huang[4], Yongle Zhan[1], Theeranan Tangthong[2], Rong Na[1*]*

[1]Division of Urology, Department of Surgery, School of Clinical Medicine, LKS Faculty of Medicine, The University of Hong Kong, Hong Kong, China

[2]Nuclear Technology Research and Development Center, Thailand Institute of Nuclear Technology (Public Organization), Nakhon Nayok, Thailand

[3]Department of Otolaryngology-Head and Neck Surgery, The Eighth Affiliated Hospital, Sun Yat-sen University, Shenzhen 518033, China

[4]Department of Urology, Ruijin Hospital, Shanghai Jiao Tong University School of Medicine, Shanghai, China

Corresponding authors email: yungna@hku.hk, narong.hs@gmail.com and theeranan@tint.or.th

Supplementary Methods

Materials. Green tea (GT) (*Camellia sinensis)* extract powder was purchased from AP Operations Co., Ltd. (Chonburi, Thailand). Zinc acetate dihydrate was acquired from Merck (Darmstadt, Germany) and distilled water was used throughout the experiments. Gallic Acid was procured from Sisco Research Laboratories Pvt. Ltd. (Maharashtra, India) and Folin & Ciocalteus Phenol was obtained from Sisco Research Laboratories Pvt. Ltd. (Maharashtra, India).

Preparation of total phenols (TP) as reducing agent and stabilizing agent. GT powder was irradiated with γ-rays by using 60Co gamma rays (Atomic Energy of Canada Limited, Ottawa, ON, Canada) at a dose rate of 8.11 kGy/h at 10 kGy under ambient temperature in air. After irradiation, GT-10 powder is used to produced polyphenol. The TP extraction process was conducted as follows: 60 mg of GT-10 powder from irradiated GT was dissolved in 10 mL of DI water and mixed with an equal volume of 50% ethanol (w/v). The solution was incubated at room temperature for 25 minutes, followed by centrifugation at 13,000 rpm and 4˚C for 10 minutes. The supernatant was

then removed, and the TP pellet was collected and dried at 60˚C for 48 hours. After extraction, the obtained TP was characterized and confirmed using UV-Vis spectroscopy.

Phenol Content Determination. The total polyphenol content (TPC) in plant extracts was measured using the Folin-Ciocalteu (F-C) method, a widely accepted assay for polyphenol quantification. This method relies on the reaction between polyphenols and the Folin-Ciocalteu reagent in an alkaline medium, facilitated by the addition of sodium carbonate. In this study, polyphenols extracted from GT leaves were analyzed using this approach. Gallic acid (GA) was used as the standard reference compound at various concentrations. The F-C reagent was prepared by dissolving 2.3 mg in 10 mL of deionized (DI) water. Sample solutions and GA standards were then mixed with the F-C reagent in a 1:1 (v/v) ratio and vortexed thoroughly to ensure uniform mixing. The resulting mixtures were incubated in the dark at room temperature for 2 hours. After incubation, the absorbance was measured using UV-Vis spectroscopy to determine the TPC. Our TP had the highest TPC of 0.42 mg/mL after the measurement.

Green synthesis of ZnO nanoparticles (ZnO NPs). The spherical-shaped ZnO NPs were prepared from zinc acetate precursor at concentration 0.2 M. A 1:1 ratio of Zn acetate precursor to TP extract was maintained by mixing 0.2 M zinc acetate with 2 mg of TP extracted from 10 kGy. The solution was vortexed thoroughly and incubated overnight at room temperature (RT) to allow complete reaction. After synthesis, the resulting ZnO NPs were analyzed using UV-Vis spectroscopy.

Synthesis of ZnO spiky nanoparticles (ZnO SNPs). The spiky-shaped ZnO NPs were synthesized using a modified precipitation method based on a Dang and teams procedure reported [1]. Zinc acetate dihydrate [$Zn(CH_3COO)_2 \cdot 2H_2O$] and sodium hydroxide (NaOH) served as precursor materials. First, $Zn(CH_3COO)_2 \cdot 2H_2O$ was dissolved in 50 mL of deionized water to prepare a 0.06 M solution. Then, NaOH 0.12 M dissolved in 50 mL of distilled water was gradually added dropwise, and the TP extracted aqueous solution was mixed with the aqueous solution under continuous stirring. The pH of the solution was monitored using a handheld pH meter (Sanxin Instrument, China) and adjusted to 10. To ensure complete reaction, the mixture was stirred continuously for 5 h. The resulting precipitate was washed three times with deionized water and then transferred to a crucible for calcination at 450°C for 5 h in an annealing furnace. Finally, the obtained ZnO SNPs powder was dried and

stored for further applications. After extraction, the obtained TP was characterized and confirmed using UV-vis spectroscopy (Agilent Technologies, Santa Clara, CA, United States).

Characterization of ZnO NPs and ZnO SNPs. To determine the chemical structure of functional ZnO NPs, Fourier Transform Infrared (FT-IR) spectroscopy was conducted using a Tensor 27 Bruker spectrophotometer (Bruker, Billerica, MA, United States). FT-IR spectra were recorded with 32 scans at a resolution of 2 cm^{-1} within the frequency range of 4000–400 cm^{-1}. X-ray diffraction (XRD) measurements were performed using a Diffractometer D8 ADVANCE (Bruker, Billerica, MA, United States) with a copper radiation source (Cu Kα) at a wavelength of 1.5418 Å, operating at 40 kV and 25 mA. The XRD patterns were analyzed in the 2θ range of 20° to 70° at a step rate of 0.02°. UV-Vis absorption spectra were recorded using a Thermo Scientific Evolution 300 UV-Vis spectrophotometer with a 1-cm path length quartz cuvette. Hydrodynamic diameters and zeta (ζ) potentials were measured using a Zetasizer Nano ZS (Malvern Instruments Ltd., Malvern, United Kingdom). Transmission Electron Microscopy (TEM) imaging was conducted using a JEOL JEM 1400 TEM (Japan Electron Optics Laboratory, Akishima, Tokyo, Japan) operating at 120 kV.

Preparation of FIT-C ZnO SNPs 10 mg of ZnO SNPs were dissolved in 20 ml of deionized water with constant stirring for 1 hr. The pH of the solution was adjusted to 6 using HCl, after 3 mg FITC solution (in DMSO) was added. The solution was then stirred overnight in the dark and was centrifuged at 3000 rpm at 4 °C for 30 min. Then, the supernatant was discarded, and centrifugation was repeated until no FITC in the supernatant. To validate successful FITC conjugation, absorbance from 400 to 700 nm was measured using a spectrophotometer. Then, ZnO SNPs -FITC were suspended in deionized water for imaging analyses and conjugation analysis using flow cytometry.

Preparation of ZnO NPs and ZnO SNPs for cellular phenotypes experiment. Total phenols extracts and Zn acetate synthesized from previous methods were separately dissolved in 3ml of deionized water (vortex vigorously). Then, TP extracts solution and Zn acetate solution were mixed gently in light-protected container (vortex vigorously for 10 seconds). The mixture was then incubated at room temperature (in dark environment) for 12-15 hours to form ZnO NPs solution. After the incubation, the dark brown precipitate revealed the formation of ZnO NPs. The mixture was then stirred for 30 minutes or sonicated at 25 °C for 15 minutes for equal distribution of the particles. Then, the solution of ZnO NPs were diluted in various concentrations of 0, 6.25, 12.5, 25, 50 and

100 μg/mL before subsequent cellular experiment. For ZnO SNPs, dried ZnO SNPs powder was diluted in deionized water to various concentrations of 0, 6.25, 12.5, 25, 50 and 100 μg/mL before subsequent cellular experiment.

***In vitro* validation**

Cell culture. Four human prostate cancer (PCa) cell lines (PC-3 RRID: CVCL_0035, DU-145 RRID: CVCL_0105, LNCaP RRID: CVCL_0395, 22RV1 RRID: CVCL_4Y35), three human thyroid cancer cell lines (TPC-1 RRID: CVCL_6298, BCPAP RRID: CVCL_0153, 8305C RRID: CVCL_1053) and Benign Prostatic Hyperplasia (BPH-1 RRID: CVCL_109) were purchased from Cell Bank of Chinese Academy of Sciences (Shanghai, China). They were cultured in Roswell Park Memorial Institute medium (RPMI 1640) (Gibco, Grand Island, NY, United States) with 10% heat-inactivated fetal bovine serum (FBS) and 1% antibi-otic-antimycotic (ThermoFisher Scientific, Waltham, MA, United States). All cell lines were cultured in Roswell Park Memorial Institute medium (RPMI 1640) (Gibco, USA) with 10% heat-inactivated fetal bovine serum (FBS) and 1% antibiotic-antimycotic (ThermoFisher Scientific). All cell lines were incubated in a humidified incubator at 37 °C with 5% CO_2 supply and were passaged once every 2–3 days using trypsin and PBS (washing process). Cells were regularly tested for Mycoplasma contamination using service from Centre for PanorOmic Sciences (CPOS) provided by Li Ka Shing Faculty of Medicine under The University of Hong Kong throughout the cell culture process.

Methyl Thiazolyl Tetrazolium (MTT) Assay. Cell proliferation was determined using Cell Proliferation Kit I (MTT) (Roche, Basel, Switzerland) stored at -20 °C. Cells were seeded at a population density of 2000 cells/well for PC-3, DU-145, 8305C and 4000 cells/well for LNCaP, 22RV1, BPH-1, TPC-1 and BCPAP respectively in 96-well plates. Cells were treated with various concentrations of ZnO NPs and ZnO SNPs (0, 6.25, 12.5, 25, 50 and 100 μg/mL) for 48 hrs. Then, the cells were treated with 10 μL of MTT labeling reagent (5mg/ml) for 4 hrs at 37 °C. After that, MTT and medium were completely aspirated and 100 μL dimethyl sulfoxide (DMSO) (Sigma-Aldrich, St. Louis, MO, United States) (Sigma-Aldrich, USA) was added for 15 min at 37 °C. Absorbance at 570 nm was measured by Thermo Varioskan flash reader. The statistical analysis was performed in GraphPad Prism 10 using Dose-response curve to calculate percent cell viability and half-maximal inhibitory concentration (IC_{50}) of ZnO NPs and ZnO SNPs. The experiment is performed in both internal and external triplicates.

Transwell migration and invasion assay. Assays were performed in 24-well transwell chamber with uncoated porous filters (pore size, 8 μm; Corning, NY, USA). For migration assay, 3×10^5/ml of PC-3, DU-145 and 8305C cells 5×10^5/ml of LNCaP, 22RV1, TPC-1 and BCPAP in serum-free medium were seeded into the upper chamber of each insert, and complete medium (with 10% FBS) was added to the lower chamber. For invasion assay, the upper transwell chambers were coated with 1:1 ratio media-dilute matrigel (Corning, NY, USA). Following incubation at 37°C for 12 h, non-migrated and non-invaded cells were removed from the upper surface of the filters using cotton swabs, and those that had migrated and invaded to the lower surface of the filters were fixed in ice-cold 100% methanol at room temperature for 15-30 min with air dry, followed by staining with 0.2% crystal violet at room temperature. Numbers for migrated and invaded cells are determined by counting the average number of cells per microscopic field using an inverted light microscope (magnification, x100).

Cell cycle analysis. Flow cytometry was utilized to analyze the percentage of each cell cycle phase (G1, S and G2/M). After the treatment, cancer cells were washed by PBS and trypsinized. Then, cells were collected and fixed with 70% cold ethanol at 4 °C for 1h and were centrifuged at 1500 g for 5 min and incubated in 0.5ml PBS containing 0.1mg/ml RNase for 30 min at 37 °C. stained with 50 μg/ml Propidium Iodide (Invitrogen, Carlsbad, CA, United States) were added for DNA staining Data acquisition and analysis were performed in Cell Analyzer Agilent NovoCyte Quanteon using service from CPOS. A total of 1×10^4 events in 100 ul was set for each sample. The gating and data analysis were performed using NovoExpress software and GraphPad Prism.

Caspase-3 activity Assay. Caspase-3 activity was detected using a Caspase-3 Activity Colorimetric Assay Kit (Elabscience, Wuhan, China) according to the manufacturer's protocols. After treatment and intervention, cells were cultured, digested with trypsin, and centrifuged at 500 g for 5 min before the supernatant was discarded. The pellet was resuspended with pre-cooled Cell Lysis Buffer according to the ratio of 100 μL of Cell Lysis Buffer per 1 million cells and incubated on an ice bath for 30 min. The lysed samples were centrifuged at 11000×g for 10-15 min at 4°C. The supernatant was transferred to a new tube and placed on ice for use. Lysate supernatant containing 100 μg of protein was used to configure a reaction system as described by the manufacturer's instructions. After the addition of Ac-DEVD. pNA and mixing, the reaction was incubated at 37 °C for 2–4 h. Then, the absorbance values of the samples were measured using a microplate reader at 405 nm. The calculation of the Caspase-3 activity in each group was determined by using the formula $(OD_{sample} – OD_{blank})/(OD_{control} – OD_{blank})^* 100$.

Total Cholesterol Assay. The levels of total cholesterol were determined using Total Cholesterol Assay Kit (Yeasen Biotechnology, Shanghai, China) according to the manufacturer's protocols. Briefly, after treatment and intervention, culture media was then centrifuged at 10,000 g, 4℃ for 10 min, take the supernatant and place it on ice for testing. The visible spectrophotometer/ELISA reader was preheated for more than 30 min, adjust the wavelength to 500 nm, and zero the spectrophotometer with distilled water. 50 μmol/mL cholesterol standard solution with the extract to obtain 2.5, 2, 1.25, 0.625, 0.3125, and 0.15625 μmol/mL standard solutions for use. Each standard tube in the experiment requires 20 μL of standard solution. 20 μL of samples were added 96-well plate with 180 μL working fluid followed by mix thoroughly and incubate at 37℃ for 15 min, and measure the absorbance at 500 nm. The blank tube and standard curve only need to be measured 1-2 times. The sample concentrations in μmol/mL are calculated according to the standard curve from known standard solutions.

3D spheroid generation model. Complete media for PCa cell lines for 3D spheroid formation includes RPMI 1640 (Gibco, USA), N2 supplement (Gibco, USA), B-27™ Supplement (50X) minus vitamin A (Gibco, USA), L-Glutamine (Gibco, USA), 100 ng/ul Recombinant Human Epidermal Growth Factor (EGF) (Gibco, USA), 100 ng/ul Recombinant Human Fibroblast Growth Factor-basic (FGF) (Gibco, USA). Following standard cell culture protocol, cancer cell lines (PC-3, DU-145, LNCaP, 22RV1, TPC-1, BCPAP and 8305C) spheroid sizes were standardized using Aggrewell^{400} 24 well plates (Stemcell Technologies). To achieve 1000 and 2000 cells per microwells, 1.2 x 10^6 for PC-3, DU-145 and 8305C and 2.4 x 10^6 cells for LNCaP, 22RV1, TPC-1 and BCPAP were seeded per well respectively. To do so, Aggrewell wells plates were pretreated with 500 μL of Anti-Adherence Rinsing Solution to each well to be used, centrifuged at 1300 x g for 5 minutes. Then, Anti-Adherence Rinsing Solution was aspirated, and wells were washed with 2 mL of warm RPMI 1640 basal medium. 1 mL of warm complete medium to each well was added.

ZnO NPs and ZnO SNPs treatment in cancer spheroids. After 48 hours of cell seeding, the spheroids were observed under microscope for uniformed size and morphology (scale bar = 200 um) then treated with half-maximal inhibitory concentration (IC_{50}) of ZnO NPs and ZnO SNPs for 48 hrs. Then, the size and morphology of the spheroids were observed. Spheroid are also harvested and passages for other subsequent phenotypes.

Cholesterol manipulation. The cells were washed by PBS twice and then changed to RPMI 1640 medium containing 50 μg/mL water-soluble cholesterol (CHO) (Sigma-Aldrich, USA) with or without ZnO SNPs treatment for 2 h. Then the cells were collected to perform the subsequent experiments.

Reactive Oxygen Species (ROS) Assay. The levels of intracellular ROS were determined using a Reactive Oxygen Species Assay Kit (Beyotime, China). After ZnO NPs and ZnO SNPs for 48 hrs and/or with or without cholesterol manipulation, cells were incubated with 10 μM H2DCFDA (Beyotime Biotech, China) in the dark for 20-30 min and then washed with deionized water 3 times. The fluorescence intensity was read at 525 nm and 580 nm, respectively, using a Microplate Reader (BMG CLARIOstar Plus). The data were analyzed as relative fold change of ROS.

***In vivo* toxicity evaluation.** In vivo toxicity evaluation. Male BALB/c nude mice (4–6 weeks) were maintained in an specific-pathogen-free (SPF) and access to food and water. The BALB/c nude mice were obtained from Zhuhai Bestest Biotechnology Co., Ltd. The mice were raised and maintained at Shenzhen Huarui Model Biotechnology Co., Ltd. The experimental protocol was approved by the Institutional Animal Care and Use Committee (IACUC) of Shenzhen Huarui Model Biotechnology Co., Ltd with HKU-SZ hospital (China); No. APS-260224-0038-001 (Approval date: 15 April 2026) in accordance with the Chinese National Standard Guideline for Ethical Review of Animal Welfare (GB/T 35892-2018). After 14 days of ZnO NPs and ZnO SNPs treatment, the mice were enthanized. Major organs (liver, kidneys, spleen, lungs) were collected, Formalin-Fixed, Paraffin-Embedded (FFPE) to perform Hematoxylin and Eosin Staining (H&E) for histopathogical observation for toxicity.

Cholesterol depletion and rescue. For Cholesterol depletion, PC3 and 8305C were supplemented with 10% Lipoprotein-Deficient Serum (LPDS) in RPMI1640 for 72 hours with subsequent ZnO NPs and Zno SNPs treatment for 48 h. For cholesterol rescue, the cells were washed by PBS twice and then changed to RPMI 1640 medium containing 50 μg/mL water-soluble cholesterol (CHO) (Sigma-Aldrich, USA) with or without ZnO SNPs treatment for 2 h. Then the cells were collected to perform MTT assay for % cell viability. All data are expressed as the mean ± standard deviation (SD) from three independent experiments (n=3).

Cellular internalization and location of FITC-conjugated ZnO NPs and ZnO SNPs in PCa and TC cell lines.

FITC-conjugated ZnO NPs and ZnO SNPs were used to treat 2 cell lines including PCa (PC3) and TC

(8305C) for 48 hrs and cellular internalization were observed using confocal Microscope LSM 880 with Airyscan 1. ZnO NPs and ZnO SNPs (green) in in nuclear region (DAPI; blue)

Statistical analyses. Analyses were performed with GraphPad software 10 (GraphPad Software, CA, USA) from at least three independent experiments, presented as the mean ± standard deviation (SD). Statistical analyses. GraphPad software 10 (GraphPad Software, CA, United States) was used as a tool to perform analyses from at least three independent experi-ments with the mean ± standard deviation (SD). Kruskal-Wallis statistic (non-parametric test) was used to analyze differences in each experimental group and p value < 0.05 was considered statistically significant. (**** $p < 0.0001$; *** $p < 0.001$; ** $p < 0.01$; *$p < 0.05$; ns, no significant difference).

[1] Z. Dang, J. Sun, J. Fan, J. Li, X. Li, T. Chen, *Materials Science and Engineering: C* **2021**, *124*, 112071, https://doi.org/https://doi.org/10.1016/j.msec.2021.112071.
